# Transient DNMT3L Expression Reinforces Chromatin Surveillance to Halt Senescence Progression in Mouse Embryonic Fibroblast

**DOI:** 10.3389/fcell.2020.00103

**Published:** 2020-03-04

**Authors:** Yoyo Chih-Yun Yu, Tony ZK Hui, Tzu-Hao Kao, Hung-Fu Liao, Chih-Yi Yang, Chia-Chun Hou, Hsin-Ting Hsieh, Jen-Yun Chang, Yi-Tzang Tsai, Marina Pinskaya, Kai-Chien Yang, Yet-Ran Chen, Antonin Morillon, Mong-Hsun Tsai, Shau-Ping Lin

**Affiliations:** ^1^Institute of Biotechnology, National Taiwan University, Taipei, Taiwan; ^2^Department of Microbiology and Immunology, University of British Columbia, Vancouver, BC, Canada; ^3^Canada’s Michael Smith Genome Sciences Centre, British Columbia Cancer Agency, Vancouver, BC, Canada; ^4^Center for Systems Biology, National Taiwan University, Taipei, Taiwan; ^5^ncRNA, Epigenetic and Genome Fluidity, CNRS UMR 3244, Sorbonne Université, PSL University, Institut Curie, Centre de Recherche, Paris, France; ^6^Graduate Institute and Department of Pharmacology, National Taiwan University School of Medicine, Taipei, Taiwan; ^7^Agricultural Biotechnology Research Center, Academia Sinica, Taipei, Taiwan; ^8^Research Center for Developmental Biology and Regenerative Medicine, Taipei, Taiwan

**Keywords:** senescence, epigenetics, DNA methyltransferase 3-like (DNMT3L), polycomb repressive complex 2 (PRC2), chromatin surveillance, transposable element (TE)

## Abstract

Global heterochromatin reduction, which is one of the hallmarks of senescent cells, is associated with reduced transposable element repression and increased risk of chromatin instability. To ensure genomic integrity, the irreparable cells in a population exit permanently from the cell cycle, and this process is termed “senescence.” However, senescence only blocks the expansion of unwanted cells, and the aberrant chromatin of senescent cells remains unstable. Serendipitously, we found that the transient ectopic expression of a repressive epigenetic modulator, DNA methyltransferase 3-like (DNMT3L) was sufficient to delay the premature senescence progression of late-passage mouse embryonic fibroblasts (MEFs) associated with a tightened global chromatin structure. DNMT3L induces more repressive H3K9 methylation on endogenous retroviruses and downregulates the derepressed transposons in aging MEFs. In addition, we found that a pulse of ectopic DNMT3L resulted in the reestablishment of H3K27me3 on polycomb repressive complex 2 (PRC2)-target genes that were derepressed in old MEFs. We demonstrated that ectopic DNMT3L interacted with PRC2 in MEFs. Our data also suggested that ectopic DNMT3L might guide PRC2 to redress deregulated chromatin regions in cells undergoing senescence. This study might lead to an epigenetic reinforcement strategy for overcoming aging-associated epimutation and senescence.

## Background

Cellular senescence is a major response to irreversible damage acquired in response to stress or replication mistakes (d’Adda, 2008; Hernandez-Segura et al., 2018). Senescent cells exhibit permanent cell-cycle arrest and substantial chromatin remodeling (Cruickshanks et al., 2013; Tchkonia et al., 2013). The number of senescent cells in tissues increases with age (Spaulding et al., 1997; Krishnamurthy et al., 2004; Herbig et al., 2006; Krizhanovsky et al., 2008; Vidal et al., 2012; Waaijer et al., 2012), and the rate of their accumulation predicts the lifespan of mice (Jurk et al., 2014). The secretions from senescent cells lead to a senescence-associated secretory phenotype (SASP), which might contribute to aging-associated tissue dysfunction or the development of a cancerous niche in old tissue (Coppe et al., 2010; Castro-Vega et al., 2015; Buhl et al., 2019; Lopes-Paciencia et al., 2019). A recent study showed that the transplantation of senescent cells into mice leads to the early onset of aging-related phenotypes (Xu et al., 2017, 2018), which supports the current hypothesis that senescence can be a driver of aging (Krtolica et al., 2001; Sturmlechner et al., 2017; Lewis-McDougall et al., 2019).

Emerging studies suggest the elimination of senescent cells to extend the healthspan (Baker et al., 2011, 2016; Xu et al., 2015; Palmer et al., 2019). The Kirkland research group initiated a quest for specific activation of the programed death of senescent cells (Zhu et al., 2015), which has resulted in interest in the identification of various senolytic compounds or drugs that can kill senescent cells of specific cell lineages (Zhu et al., 2017; Fuhrmann-Stroissnigg et al., 2018; Cherif et al., 2019; Justice et al., 2019). In addition, it has been found that several emerging senomorphic drugs can suppress SASP (Bitto et al., 2016; de Cecco et al., 2019). The current senotherapeutic interventions basically focus on pre-existing senescent cells (Myrianthopoulos, 2018; Kim and Kim, 2019), whereas the process for safely slowing the emergence of senescence is poorly understood.

Senescence can be activated by reaching the Hayflick limit (telomere shortening to a critical length) (Hayflick and Moorhead, 1961; Hayflick, 1965) or stressors (Serrano et al., 1997; Wang et al., 2009; Luo et al., 2011; Suram et al., 2012; de Magalhaes and Passos, 2018). In addition to the continuous attrition of telomere length accompanied by cell division, aging cells approaching senescence display a less-stringent chromatin architecture and epigenetic control (Pal and Tyler, 2016; Xie et al., 2018; Benayoun et al., 2019), and these features can be accelerated by stress induced by stochastic replication mistakes, environmental stimuli, oncogene activation or signals produced by pre-existing senescent cells (Hodny et al., 2010; Nelson et al., 2012; Benayoun et al., 2015). Therefore, many cells enter senescence before reaching the Hayflick limit (Spaulding et al., 1997; Rubin, 2002; Toussaint et al., 2002; Parrinello et al., 2003; Hewitt et al., 2012; Suram et al., 2012), and this process is known as premature senescence.

The gradual loss of chromatin organization during aging is associated with perinuclear-chromatin detachment (associated with the depletion of the nuclear-envelope protein lamin B1) (Guelen et al., 2008; McCord et al., 2013), reductions in histone core proteins (H3 and H4) (O’Sullivan et al., 2010), and changes in trimethylation markers on histone H3 lysines (H3K4me3, H3K9me3, and H3K27me3) resulting from the reconfiguration of chromatin regulators during aging (Pal and Tyler, 2016). These features contribute to a decline in the maintenance of heterochromatin (inactivated, condensed chromatin state), which is a hallmark of senescent cells (de Cecco et al., 2013a; Pal and Tyler, 2016).

Heterochromatin is mainly concentrated on repeat sequences, including transposable elements (TEs), which constitute a major fraction of the mammalian genome (Lander et al., 2001; Mouse Genome Sequencing Consortium et al., 2002). TE derepression is frequently observed during cellular aging and cancer development (Belancio et al., 2010; de Cecco et al., 2013a, b, 2019). The reactivation of TEs in somatic cells reflects the increases in heterochromatin loss and global chromatin relaxation observed with aging (Orr, 2016; Pal and Tyler, 2016). Elevated chromatin accessibility leads to genomic instability, which can serve as a prelude to senescence. Therefore, the enhancement of chromatin surveillance might allow prolongation of the healthspan of a cell, and the appropriate perturbation of chromatin modifiers reportedly extends the lifespan of invertebrate models (Pal and Tyler, 2016; Sen et al., 2016; Benayoun et al., 2019; Mahmoudi et al., 2019).

Serendipitously, we discovered that the transient ectopic expression of a repressive epigenetic modulator, DNA methyltransferase 3-like (DNMT3L), was sufficient to delay the senescence progression of late-passage mouse embryonic fibroblasts (MEFs). MEFs usually enter senescence after approximately 10 passages when cultured *in vitro* under standard cultural conditions with ambient oxygen (20%), and these cells still contain relatively long telomeres (Parrinello et al., 2003). Therefore, MEFs are considered a useful model for the study of premature senescence independent of telomere shortening (Cristofalo et al., 2000).

DNMT3L is a well-studied TE suppressor (Liao et al., 2012). In addition to the maintenance of heterochromatin obtained with endogenous DNMT3L in germ cells, we discovered that ectopic DNMT3L expression can recruit a repressive chromatin-modifying complex to stimulate *de novo* repressive histone modification markers on newly infected retroviruses and endogenous retroviruses (ERVs) in MEFs (Kao et al., 2014). This finding resonates with DNMT3L’s known function of facilitating the epigenetic repression of TE-associated regions during germ cell development after the physiological genome-wide erasure of repressive epigenetic markers (Bourc’his and Bestor, 2004; Hata et al., 2006; Hu et al., 2008).

In this study, we discovered that the transient expression of DNMT3L in MEFs is sufficient to sustain the proliferation activity of cells for at least 40 passages *in vitro* under standard 20% oxygen culture conditions. To gain insights into the mechanism underlying this phenomenon, we examined several factors associated with the aging process, including the quantities of the nuclear envelope-binding protein lamin B1, histone proteins, and repressive H3K9me3 markers on ERVs and the expression level of selected TEs. To understand the effect of DNMT3L on aging-associated single-copy genes, we further performed a cDNA microarray analysis of young, old and DNMT3L-treated MEFs and characterized the properties of DNMT3L-responsive genes that are derepressed in old MEFs. Our data suggest that chromatin surveillance enhancement might constitute one of the mechanisms underlying the DNMT3L-induced halting of senescence progression in aging MEFs. This type of study might lead to the development of an epigenetic reinforcement strategy that could mitigate aging-associated epimutation and prevent premature senescence.

## Results

### A Pulse of Ectopic DNMT3L Delays Premature Senescence in Mouse Embryonic Fibroblasts

**FIGURE 1:**
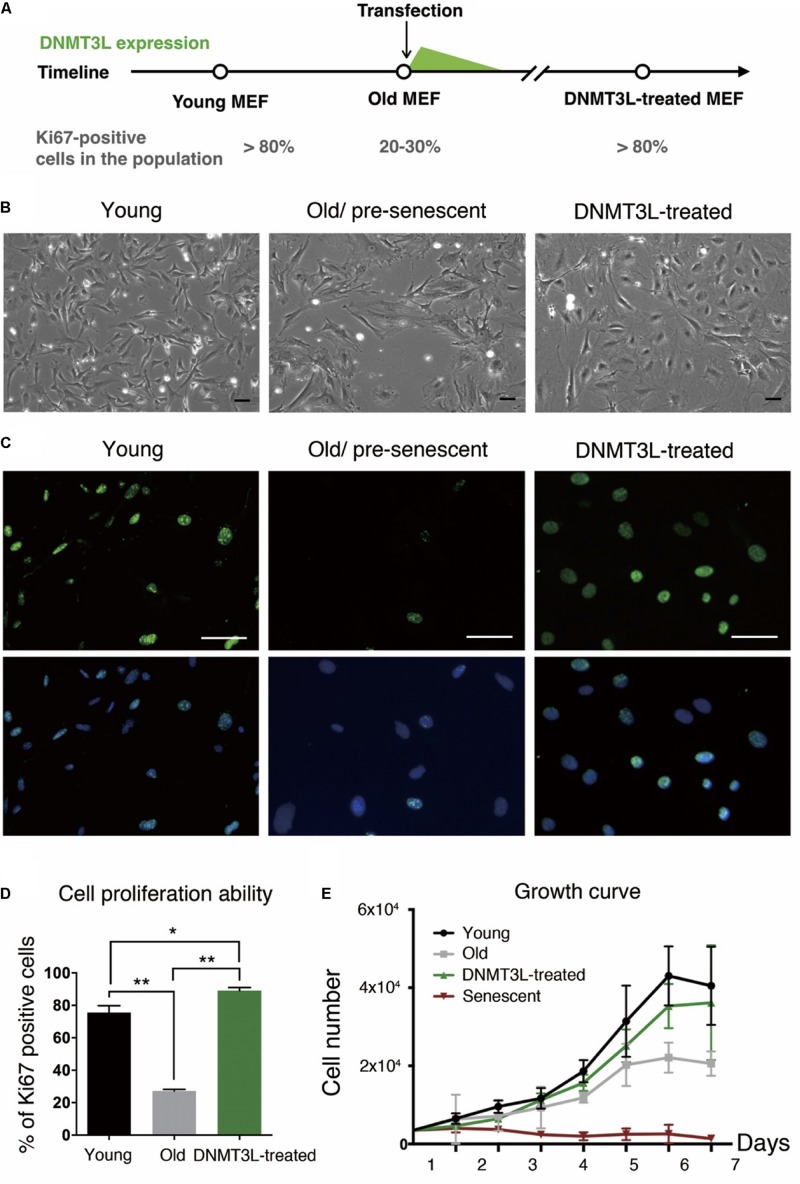
Ectopic DNMT3L pulse restored the proliferation ability of aging MEFs. **(A)** Timeline of the sampling time points and the ectopic DNMT3L expression timing. The percentage of Ki67-positive cells in the population indicated the stage of cells. **(B)** Representative bright-field microscopy images of young, old/presenescent and DNMT3L-treated MEFs as labeled. Scale bar = 10 μm. **(C)** Representative Ki67-immunofluorescence images of the indicated cells. Green: Ki67 (proliferating cells); blue: Hoechst 33342 (nuclei). Bar = 10 μm. **(D)** Frequency distribution of proliferating cells among the three cell types based on the percentage of Ki67–positive nuclei. Mean ± SEM, *n* = 3. Asterisks indicate averages with significantly differences at *p < 0.05 or **p < 0.01, as determined by Student’s *t*-test. **(E)** Growth curve of the labeled cell populations. The black lines indicate the young MEFs; the gray lines indicate the old/presenescent MEFs; the green lines indicate the DNMT3L-treated MEFs; and the dark-red lines indicate the senescent MEFs. The cells were initially seeded at 5000 per well in a 24-well tissue culture plate (mean ± SEM, *n* = 6).

Here, we demonstrated for the first time that transient DNMT3L expression in late-passage MEFs was sufficient to extend the proliferative activities of these cells. Intriguingly, transient DNMT3L expression in early passage MEFs failed to delay senescence (Supplementary Figure S1). This observation indicated that this DNMT3L-dependent resistance to senescence was restrictive to the presenescent cellular environment in MEFs. We therefore determined the expression timing of *Dnmt3l* in MEFs in a restrictive range of passages based on the percentage of Ki67-positive cells (actively dividing cells) (Figure 1A). Old/presenescent MEFs steadily proliferated after transient exposure to DNMT3L. We termed the cells after a DNMT3L pulse as “DNMT3L-treated MEFs” (the representative passages used in this paper were at least 10 passages after transient DNMT3L expression, when DNMT3L was no longer present). The DNMT3L-treated MEFs sustained robust cell division for over 40 passages and were still growing well after 40 passages, whereas MEFs transfected with a mock expression vector (used as a control) barely showed any division within five passages after the transfection. Compared with the flat and irregular shapes of old/presenescent MEFs, the DNMT3L-treated MEFs bulged in the center and had smaller nuclei (Figure 1B). Remarkably, the DNMT3L-treated MEFs showed around 80% enrichment in Ki67-positive cells, and this finding is similar to that found for young MEFs, which can be defined as 80% Ki67-positive cells (Figures 1C,D). BrdU-positive cells were also significantly enriched in DNMT3L-treated MEF (similar to that found in young MEFs) comparing with old/presenescent MEFs, indicating active DNA replication after the DNMT3L pulse (Supplementary Figure S2). The growth curve of the DNMT3L-treated MEFs was closer to that of young MEFs than to that of old/presenescent MEFs and was clearly different from that of senescent MEFs (Figure 1E). The Ki67 index and growth curve of the DNMT3L-treated MEFs suggested that transient DNMT3L expression restored the proliferative ability of old/presenescent MEFs.

### The DNMT3L-Induced Halting of the Senescence Machinery Might Be Partly Due to Maintenance of the Nuclear Architecture

**FIGURE 2:**
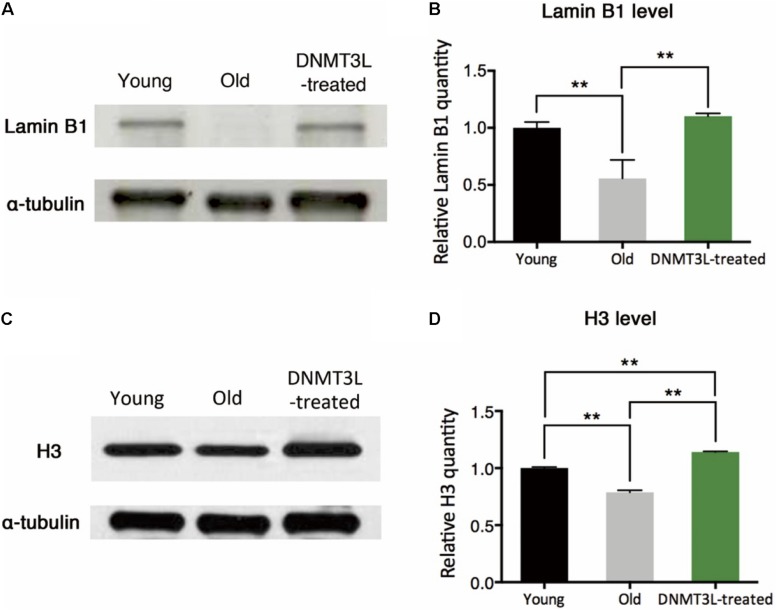
DNMT3L treatment restored the nuclear envelope-binding and histone core proteins that decreased during cellular aging. **(A)** Western blot analysis of extracts from young, old/presenescent and DNMT3L-treated MEFs using anti–lamin B1 and anti–α-tubulin antibodies. The distribution of the α-tubulin quantity was relatively stable among populations of young, old/presenescent and DNMT3L-treated MEFs with the same cell number. **(B)** Statistical results from repeated immunoblots assessing the relative quantity of lamin B1 normalized by α-tubulin. Mean ± SEM, *n* = 3. Asterisks indicate averages with significant differences at **p < 0.01, as determined by Student’s *t*-test. **(C)** Western blot analysis of extracts from the same set of cell lines using anti–H3 and anti–α-tubulin antibodies. **(D)** Statistical results from repeated immunoblots assessing the relative quantity of H3 normalized by α-tubulin. Mean ± SEM, *n* = 3. Asterisks indicate significant differences at **p < 0.01, Student’s *t*-test.

The quantity of the nuclear envelope-binding protein lamin B1 and the associated heterochromatin usually declines during the aging process, and these decreases are associated with reductions in the total nucleosome numbers. The transient treatment of aging MEFs with DNMT3L not only induced long-term proliferation activity but also significantly restored the lamin B1 and H3 expression levels (Figures 2A–D), which suggested reinforcement of the condensed global chromatin structure. These results indicated that the transient expression of DNMT3L in presenescent MEFs enabled the halting of senescence progression partly via the maintenance of sufficient levels of nuclear lamina protein and nucleosomes.

### A Pulse of Ectopic DNMT3L Enhances H3K9me3 on ERVs in a Long-Term Manner

**FIGURE 3:**
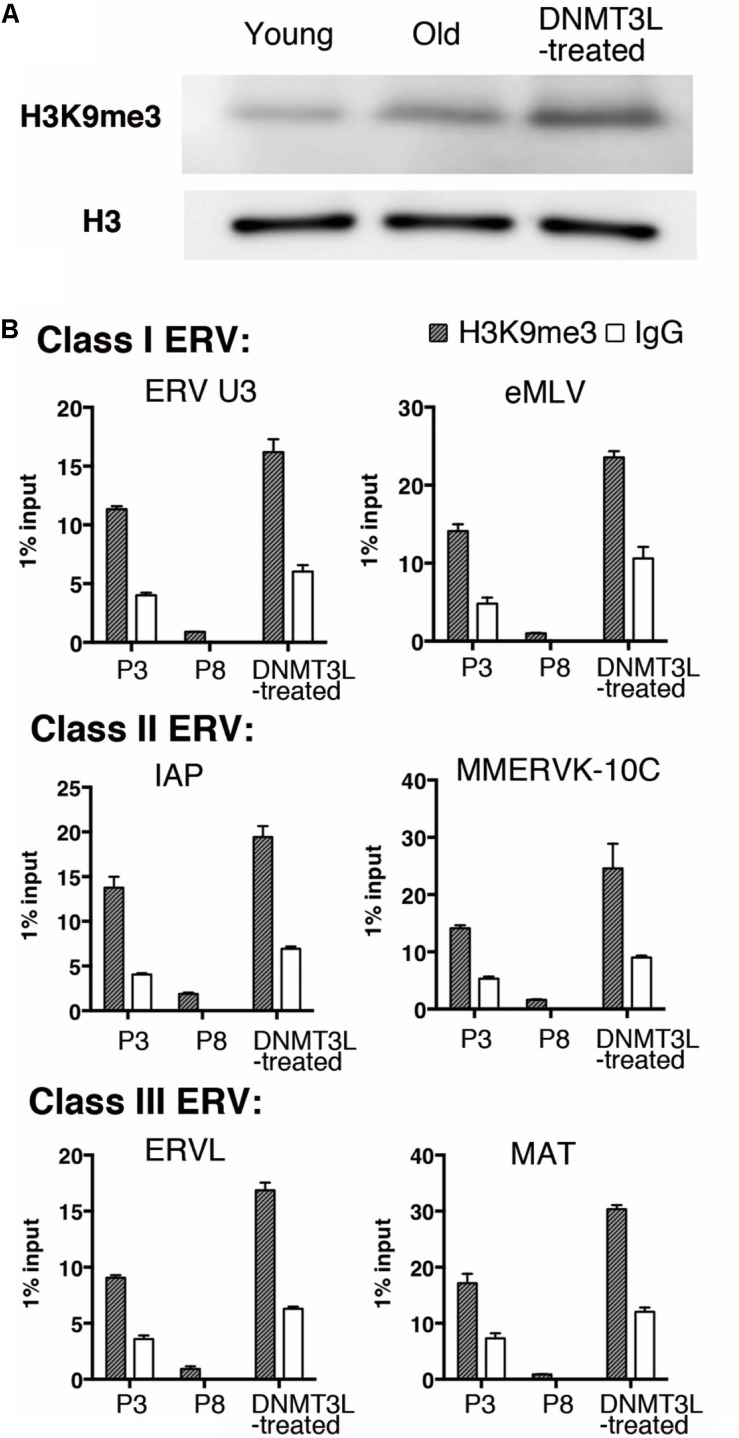
Ectopic DNMT3L established a long-term effect on global H3K9me3 elevation and H3K9me3 enrichment on ERVs. **(A)** Western blot analysis of extracts from young, old/presenescent and DNMT3L-treated MEFs using anti–H3K9me3 antibody. Anti–H3 antibodies were used to indicate the relative expression on nucleosomes among samples. **(B)** ChIP-qPCR with anti-H3K9me3 antibody was used to assess the enrichment of H3K9me3 on representative Class I to Class III ERVs in the indicated cells. Mean ± SEM, *n* = 3. Rabbit IgG was used as a negative control.

We previously found that the H3K9me3 marks on two TE families, Class I and II ERVs, were significantly elevated in DNMT3L-expressing MEFs at 48 hr post-*Dnmt3l* transfection (Kao et al., 2014). After this immediate response, we found a global elevation of H3K9me3 in DNMT3L-treated MEFs (Figure 3A) and a long-term maintenance of H3K9me3 enrichment in all three classes of ERVs in DNMT3L-treated MEFs for at least 10 passages after a DNMT3L pulse administered once ectopic DNMT3L was no longer detectable (Figure 3B). In addition, most of the selected TE subfamilies included in the RT-qPCR analysis were derepressed in old MEFs compared with their expression in young MEFs. The great majority of the derepressed TE subfamilies were downregulated after DNMT3L treatment (data not shown). Although DNMT3L protein is not detectable in MEFs, the above findings suggested a long-term effect carried over from prior ectopic DNMT3L expression.

### DNMT3L Treatment Partly Reverses Aberrant Gene Expression During the Aging Process in MEFs

To understand the impacts of DNMT3L exposure on single-copy genes in old MEFs, we compared the annotated gene expression patterns of young, old/presenescent and DNMT3L-treated MEFs through a microarray analysis. The pairwise Spearman correlation matrix showed that the gene expression levels were similar between young and old MEFs (Supplementary Figure S3). The genes upregulated in young compared with old MEFs were largely associated with proliferation-related pathways (Supplementary Figure S4a), whereas the genes upregulated in old MEFs were enriched in other pathways and included genes negatively regulated by Sirt1, genes associated with oxidative stress-induced senescence, and genes associated with SASPs (Supplementary Figure S4b). Intriguingly, a significant proportion of the genes that were differentially expressed between young and old MEFs were also affected by DNMT3L treatment. We found 1006 differentially expressed genes (DEGs) between young and old MEFs, and 556 of these DEGs also showed differential expression before and after the DNMT3L pulse (Supplementary Figure S5). The hypergeometric *p*-value of this overlap was less than 2 × 10*^–^*^16^, which suggested that DNMT3L exerted a significant impact on genes that exhibit altered expression among passages. We found that DNMT3L treatment partly reversed the cellular aging-related transcriptome changes in MEFs during the aging process. Among the 556 genes whose expression was affected by both prolonged passages and DNMT3L treatment, 82.7% exhibited a young cell-like expression pattern after DNMT3L treatment (Supplementary Figures S6a–d). Intriguingly, the majority of the genes upregulated in old MEFs were re-repressed after DNMT3L treatment. We also performed proteomic analysis on the young, old, and DNMT3L-treated MEFs. The changes in the protein expression patterns were highly associated with the DEGs identified in the microarray analysis (data not shown).

### DNMT3L Enhances the Repression of Derepressed PRC2-Targeted Genes in Old MEFs and Globally Reinforces H3K27me3 Markers

**FIGURE 4:**
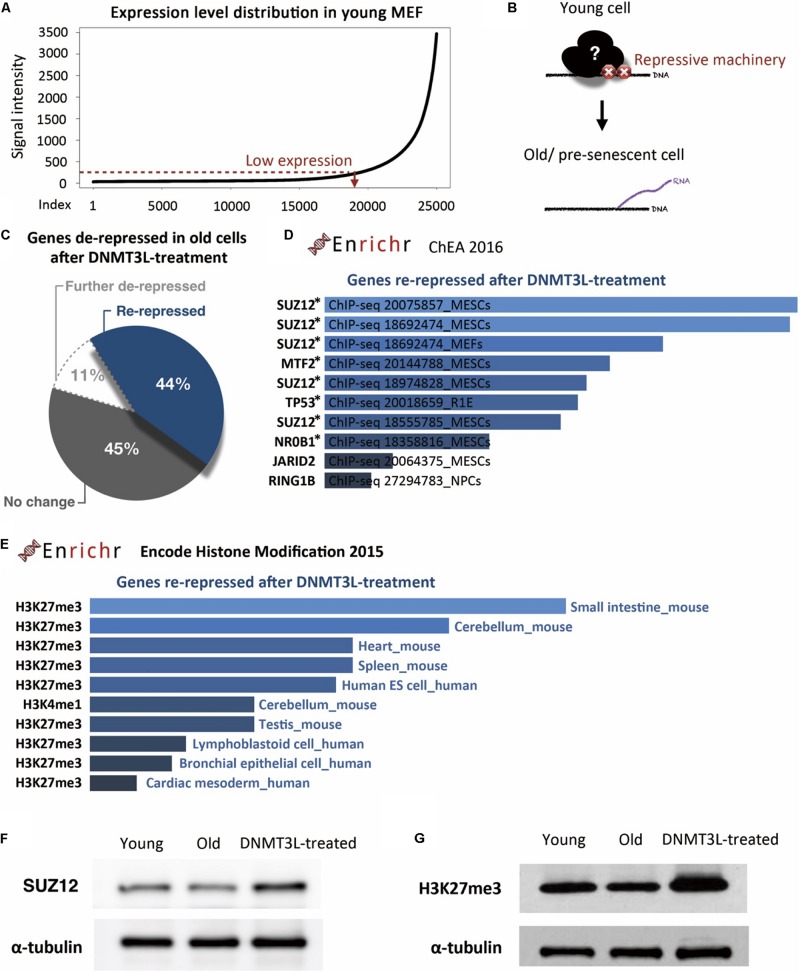
DNMT3L treatment enhanced the repression of derepressed PRC2-targeted genes in old MEFs and globally reinforced H3K27me3 markers. **(A)** The distribution of gene expression in young MEFs was determined by a microarray analysis. According to the curve, we manually defined a threshold for the probe signal intensity below 100 as indicating a gene with low expression. The following analysis focused on these low-expression genes that lose repression in old MEFs. **(B)** Illustration of the genes with aging-associated loss of repression and their original repressor in young MEFs. **(C)** Pie chart indicating the change in genes derepressed in old MEFs after DNMT3L treatment. **(D)** The top 10 potential consensus transcriptional regulators of genes derepressed in old MEFs and re-repressed after a DNMT3L pulse are listed. The gene set described above was submitted to Enrichr and aligned with the ChEA 2016 database. All the listed regulators, SUZ12, MTF2, TP53, NR0B1, JARID2, and RING1, have significant hits with *p* < 0.05. The asterisks indicate the genes with *p*-values < 0.05. **(E)** Most-consensus histone modification of the genes described above in other cell lineages according to Encode Histone Modification 2015 (Enrichr). The listed markers in each dataset have a significance of *p* < 0.05. **(F,G)** Western blot analysis of extracts from young, old/presenescent and DNMT3L-treated MEFs with anti-SUZ12 and anti-H3K27me3 antibodies. The quantity of α-tubulin represents the loading control of similar cell numbers.

To identify the repressive machinery lost during aging, we focused on the genes repressed in young MEFs and derepressed in old MEFs. We first defined a set of genes with expression levels below a manually positioned cutout line in young MEFs (Figure 4A). These genes represented those that properly maintained the repressed/silenced machinery at early passages (Figure 4B). Among the 19,116 genes that were expressed at low levels in young MEFs, we then spotted 419 hits that were overexpressed by more than 1.5-fold in old MEFs.

Among these 419 hits, only 11% were continuously upregulated after DNMT3L treatment, and half of the remaining 89% were downregulated after the treatment (Figure 4C). This distribution suggested that ectopic DNMT3L mainly played a repressive role on the genes derepressed in old MEFs, resulting in establishment of a “young cell-like” pattern, which is consistent with the primarily repressive function attributed to DNMT3L.

According to ChIP Enrichment Analysis (ChEA) 2016 (a gene list enrichment analysis tool from Enrichr), SUZ12, a PRC2 member responsible for establishment of the H3K27me3 repressive marker, showed a significantly higher potential to be one of the binding factors for genes re-repressed after DNMT3L treatment. MTF2, a polycomb group (PcG) protein that recruits PRC2 to induce an enhancement in H3K27me3 methylation activity, was also in the list of top five proteins with a significant adjusted *p*-value (Figure 4D). The microarray data showed that the RNA expression levels of *Suz12*, *Mtf2* and *Trp53* were downregulated in old MEFs compared with those in both young and DNMT3L-treated MEFs, whereas *Nr0b1* and *Jarid2* maintained similar levels among all of the samples (Supplementary Figure S7).

Additionally, the above-described genes derepressed in old MEFs and re-repressed after DNMT3L treatment were subjected to Encode Histone Modification 2015 (another gene list enrichment analysis tool from Enrichr). These genes are mainly found to be marked by H3K27me3 in various mouse somatic cell lineages (Figure 4D). We performed a Western blotting analysis to measure the overall levels of SUZ12 and H3K27me3 in young, old and DNMT3L-treated MEFs (Figure 4E). SUZ12 was slightly downregulated in old MEFs and upregulated in DNMT3L-treated MEFs compared with its expression in young MEFs, which was consistent with the *Suz12* RNA expression levels obtained in the microarray analysis (Supplementary Figure S7). Among these cells, old MEFs showed the lowest levels of both H3 and H3K27me3 (Figures 2C,D, 4F,H). After normalization based on alpha-tubulin, the H3K27me3 level was globally increased in DNMT3L-treated MEFs (Figure 4F). Our data suggested that DNMT3L can enhance the repressive regulation of genes that lose H3K27me3 repression during aging and reinforce the global H3 and H3K27me3 levels.

### Ectopic DNMT3L Interacts With SUZ12 and Restores H3K27me3 in the Promoters of Derepressed PRC2 Target Genes

The above-described data support the hypothesis that DNMT3L mitigates the derepression of PRC2-target genes that showed a loss of H3K27me3 suppression during aging. Based on the global elevation of SUZ12 and H3K27me3 after DNMT3L treatment in MEFs, and as DNMT3L and EZH2 interaction has been observed in ES cells before (Neri et al., 2013), we hypothesized that DNMT3L might facilitate the re-establishment of H3K27me3 markers on derepressed PRC2 target genes.

**FIGURE 5:**
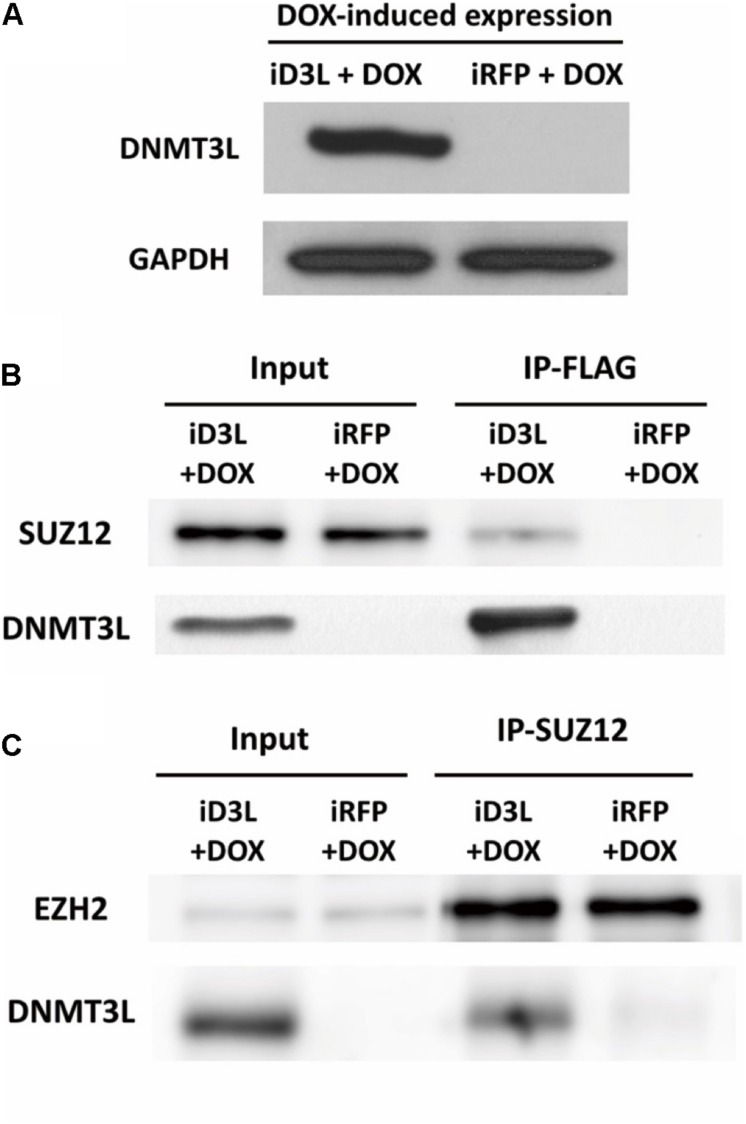
Ectopic DNMT3L interacted with the PRC2 member SUZ12. **(A)** DNM3L or RFP expression was induced by 48 h of treatment with doxycycline (DOX) in lentivirus-infected DOX-inducible MEFs expressing the manufactured gene (Supplementary Figure S6). Western blot (WB) analysis of extracts of the same numbers of DNMT3L-expressing- (iD3L + DOX) and red fluorescent protein (RFP)-expressing (iRFP + DOX) MEFs using anti-DNMT3L antibody. Anti-GAPDH antibody was used as a loading control. **(B,C)** Immunoprecipitation (IP) and WB results revealed the interaction between DNMT3L and PRC2 members. Whole-cell extracts from DNMT3L- and RFP-expressing MEFs at 48 h postinduction were subjected to IP with anti–FLAG and anti–SUZ12 antibodies. WB using antibodies against DNMT3L, SUZ12, and EZH2 was performed to detect these proteins in the indicated IP-pulldown lysate extracts. SUZ12 and EZH2 showed equal quantities after a 5% input of both DNMT3L- and RFP-expressing MEFs. The presence of DNMT3L and SUZ12 was found in each protein interaction complex.

To test whether DNMT3L can recruit PRC2, we examined whether PRC2 and ectopic DNMT3L can be precipitated in the same complex in MEFs through a coimmunoprecipitation (co-IP) assay. Due to the lack of a suitable IP-grade anti-DNMT3L antibody, we constructed a lentiviral vector encoding N-terminal FLAG-tagged DNMT3L under the control of a doxycycline-inducible promoter. Young MEFs were transduced, selected using puromycin and treated with doxycycline at the time point corresponding to the 30% Ki67 index described previously (Supplementary Figure S8a). The expression of exogenous *Dnmt3l* was well controlled by doxycycline (Figure 5A and Supplementary Figure S8b). As expected, DNMT3L coprecipitated the PRC2 member SUZ12 (Figure 5B). Consistently, the immunoprecipitation of SUZ12 with anti-SUZ12 antibody revealed the presence of DNMT3L and EZH2, another PRC2 member (as a control), in the pull-down lysate (Figure 5C). We also observed HDAC1 in the IP-SUZ12 pull-down complex in MEFs and observed enrichment of the HDAC1-SUZ12 interaction in the DNMT3L-expressing MEFs (data not shown).

**FIGURE 6:**
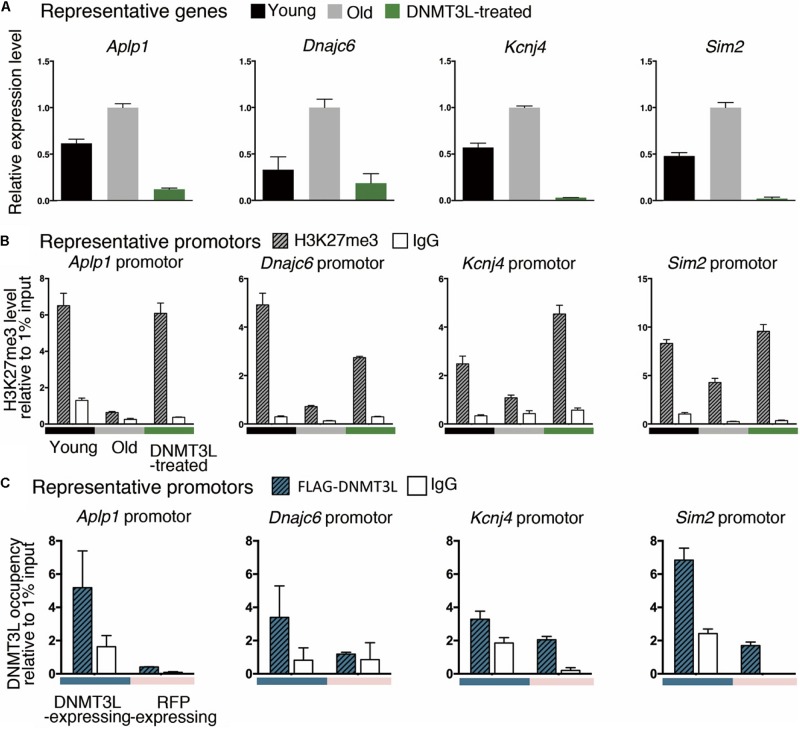
Ectopic DNMT3L pulse restored H3K27me3 on the promoter of PRC2-target genes derepressed in old MEFs. **(A)** An RT-qPCR analysis (±SEM) demonstrated the relative expression of representative genes. The gene expression levels were normalized by *Rplp0*, and three technical repeats were performed. The *y*-axis represents the expression fold-changes relative to the gene expression in old MEFs. Representative genes: *Aplp1, Dnmajc6, Kcnj4*, and *Sim2*. **(B)** Anti-H3K27me3 ChIP-qPCR was performed to demonstrate the enrichment of H3K27me3 on the promoter or exon1 of the represented genes. Negative control: mouse IgG. The RNA expression of representative genes was reciprocally correlated to the accumulation of H3K27me3 in the promoter. **(C)** The occupancy of DNMT3L on the promoter or exon 1 of the represented genes during DNMT3L expression was assessed by ChIP-qPCR with an anti-FLAG antibody. RFP-expressing MEFs served as DNMT3L-negative controls. Mouse IgG was used as the negative control for ChIP.

We randomly selected some genes from those derepressed in old MEFs and re-repressed after DNMT3L treatment (Figure 6A) and measured the H3K27me3 levels at their promoters in young, old and DNMT3L-treated MEFs through ChIP. The analysis of the genes that were re-repressed after DNMT3L treatment and showed a loss of H3K27me3 in old MEFs revealed that their H3K27me3 levels were significantly restored in DNMT3L-treated MEFs (Figure 6B). The trends found for repressive H3K27me3 among young, old and DNMT3L-treated MEFs were negatively-correlated with the mRNA abundance of each gene tested (Figures 6A,B).

We then tested whether DNMT3L re-establishes H3K27me3 in the derepressed PRC2 targets through a ChIP-qPCR assay using an anti-FLAG antibody to assess the DNMT3L occupancy on chromatin (using the doxycycline-induced DNMT3L-expressing MEFs described in Supplementary Figure S8). The signal for DNMT3L binding was slightly enriched in the tested promoters or exon1 regions in the FLAG-DNMT3L-expressing MEFs compared with that in the RFP-expressing MEFs (used as a negative control; Figure 6C). Together with the potential ability of DNMT3L to interact with PRC2, these data suggested that DNMT3L might be involved in the accumulation of H3K27me3 on the regulatory regions in genes that show a loss of PRC2 repression in old MEFs (Figures 5, 6).

**FIGURE 7:**
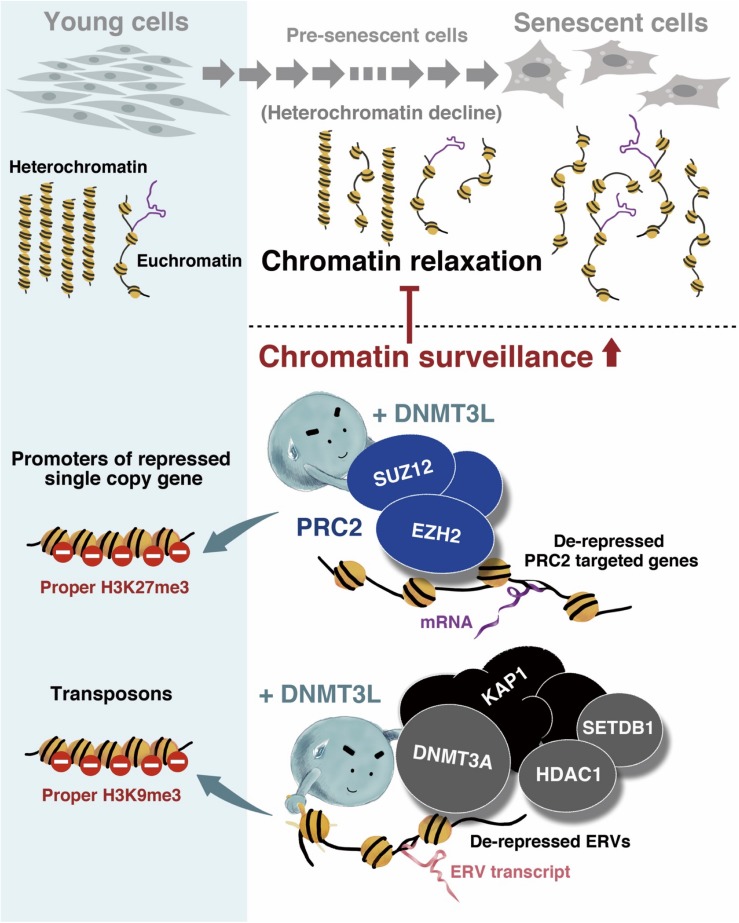
Graphical summary of the mechanism through which ectopic DNMT3L enhances chromatin surveillance and decelerates senescence progression. Global chromatin relaxation is a hallmark of aging cells. Compared with the chromatin structure in young cells, fewer heterochromatin compartments are found in aging cells approaching senescence, which consequently leads to the reactivation of transposable elements (TEs) or abnormal gene expression. The ectopic expression of DNA methyltransferase 3-like (DNMT3L) in aging MEFs enhances chromatin surveillance against heterochromatin loss during cellular aging. DNMT3L mediates repressive chromatin modulators to induce a higher amount of repressive H3K9me3 on endogenous retroviruses (ERVs) and re-establish H3K27me3 on polycomb repressive complex 2 (PRC2)-target genes that were derepressed in old MEFs. The DNMT3L pulse in aging MEFs can partially rescue chromatin relaxation and is sufficient to slow the progression of premature senescence.

In conclusion, we discovered that the transient expression of DNMT3L in old MEFs induced long-term H3K9me3 enrichment on ERV sequences and might guide the PRC2 complex to a panel of aging-associated derepressed genes and thereby introduce H3K27me3. These repressive phenomena might represent a stronger surveillance of chromatin signatures and were associated with the halting of senescence progression (Figure 7).

## Discussion

Accompanying the global relaxation of epigenetic control, an aging cell leaning toward senescence undergoes changes in its nuclear architecture, shifts in its epigenetic modifier/modification patterns, and subsequent transcriptional alterations, including TE reactivation. In this study, we demonstrated that the transient expression of DNMT3L can halt senescence progression in aging cells. It is also sufficient to restore the global core histone protein and lamin B1, which are significantly reduced in old MEFs, and to tighten up the genomic regions that show a loss of proper repression machinery in presenescent cells.

A loss of histone core proteins during aging has been observed in various species (O’Sullivan et al., 2010; Pal and Tyler, 2016; Song and Johnson, 2018), and this loss resulted in a reduction of genomic stringency. The supply of additional histone H3 and H4 partially reverses the transcriptional defects in aging cells and extends the lifespan of budding yeast (Dang et al., 2009; Feser et al., 2010). In this study, we demonstrated that the transient expression of *Dnmt3l* in presenescent cells can restore nuclear structure-related components, including global histone H3 and lamin B1. The ectopic expression of human *Dnmt3l* also elevates the expression of H3 and lamin B1 in late-passage Hs68, which are primary human foreskin fibroblasts. Consistent with our findings in MEFs, restoration after *Dnmt3l* expression was only observed in the presenescent population and not in the early passages of human fibroblasts (data not shown). Lamin B1 depletion is a remarkable feature in senescent cells. The knockdown of lamin B1 epitomizes the chromatin landscape observed in replicative senescent cells, including the emergence of heterochromatin H3K27me3-diminished regions (Shah et al., 2013) and the redistribution of the constitutive heterochromatin marker H3K9me3 (Sadaie et al., 2013).

In addition to lamin B1 restoration, we found that DNMT3L partially fixed the aberrant H3K27me3 and H3K9me3 landscape and redressed the silencing modifications in the examined gene regions. In senescent cells, transcriptional downregulation of the PRC2 member EZH2 leads to a loss of H3K27me3 and the activation of PRC2-mediated genes (Bracken et al., 2007; Maertens et al., 2009). A recent finding showed that PRC redistribution could be caused by the recruitment of PRCs to sites of DNA damage (Ginjala et al., 2011). A more comprehensive investigation combining genomic organization and functional annotation data from multiple studies linked the abnormal PRC2 signature to poised promoter and heterochromatin depletion in aging-associated regions (Dozmorov, 2015). These data highlighted PRC2 as a key regulator of age-related processes and suggested that repairing the PRC2 signature might be a novel approach against aging. Consistent with this concept, we found an association between the halting of senescence progression and the regaining of H3K27me3 markers on derepressed PRC2 targets in presenescent MEFs after transient *Dnmt3l* expression. The upregulation of SUZ12 in DNMT3L-treated MEFs may contribute to the increased accumulation of H3K27me3 (Figures 4F,G). The correction of the PRC2 signature is therefore considered at least one of the critical factors for extending the cellular healthspan.

In proliferating cells, many tumor-suppressor genes or senescence effectors are silenced by PRC2 (e.g., p16 and the *CDKN2A* gene at the INK4A locus), and in senescent cells, the transcriptional downregulation of EZH2 leads to a loss of H3K27me3 and the activation of PRC2-mediated genes (Bracken et al., 2007; Maertens et al., 2009). Although we did not observe a significant decrease in PRC2 levels in our presenescent MEFs, we found a loss of H3K27me3 in certain PRC2 targets. The previously demonstrated DNA damage induced PRC redistribution (Ginjala et al., 2011), supports our speculation of PRC2 relocalization during aging. These findings indicated that PRC2-mediated silencing might be a critical inhibitor of senescence. Additionally, the PRC2 members EZH2 and EED are known to interact with histone deacetylases (HDAC) 1 and 2 (van der Vlag and Otte, 1999; Caretti et al., 2004; Tonini et al., 2004), which suggests that based on the cellular context, transcriptional repression by the PRC2 complex might be mediated through HDACs. This result is consistent with our observation of the nuclear relocalization of HDAC1 after DNMT3L treatment (Kao et al., 2014). The cytoplasmic mislocalization of the nuclear protein HDAC1 has been described as a characteristic of cellular aging that is also linked to heterochromatin loss during aging (Willis-Martinez et al., 2010).

In addition to the DNMT3L-PRC2 activity observed in the present study, we previously found that ectopic DNMT3L expression in MEFs also triggers the formation of the DNMT3L-DNMT3A-KAP1-HDAC1-SETDB1 complex, which is linked to H3K9me3 repressive modifications at least on Class I and Class II ERVs (Kao et al., 2014), two well-known TE families. A specific point mutation of DNMT3L that breaks the DNMT3L-H3 tail or DNMT3L- DNMT3A interaction abolishes the formation of this complex (Kao et al., 2014). The interaction between DNMT3L and DNMT3A has been well demonstrated in germ cell development and stem cell differentiation. Without DNA methyltransferase activity, DNMT3L modulates chromatin by interpreting histone modifications and facilitating DNMT3A and DNMT3B for *de novo* DNA methylation (Ooi et al., 2007). The loss of DNA methylation in *Dnmt3l*-knockout (KO) male mouse germ cells leads to histone hyperacetylation and H3K9 demethylation, which consequently results in a loss of heterochromatin at the specific developmental stage of germ cells (Webster et al., 2005). According to microarray data from young, old and DNMT3L-treated MEFS, the transcription levels of *Kap1*, *Hdac1*, and *Setdb1* were lower in old MEFs and elevated in DNMT3L-treated MEFs, whereas *Dnmt3a* was expressed at similar low levels in all the samples (Supplementary Figure S9). The KAP1, HDAC1 and SETDB1 proteins in the ectopic DNMT3L-mediated repressive complex were all correlated with heterochromatin maintenance. KAP1 coordinates the assembly of a macromolecular complex containing chromatin-remodeling proteins (Sripathy et al., 2006; Iyengar and Farnham, 2011) and mediates heterochromatin-packaging repression on LINE1 (a well-known TE family). The KAP1-mediated repression of TEs fails with stress and age (van Meter et al., 2014). HDAC proteins govern heterochromatin at every cell phase (Murakami, 2013), and SETDB1 is a KAP1-associated H3K9-specific tri-methyltransferase (Schultz et al., 2002). The DNMT3L-mediated recruitment of DNMT3A-KAP1-HDAC1-SETDB1 and the elevated H3K9me3 markers on ERVs suggested the enforcement potential of heterochromatin maintenance on TEs in old MEFs (Figure 7).

The loss of heterochromatin that accompanies aging leads to a relaxed chromatin state, which potentially increases the risk of TE transposition and thereby jeopardizes the genomic stability (Pal and Tyler, 2016). In a young healthy individual, transposition rarely occurs in somatic cells. However, TE reactivation is frequently found in aging or cancer cells due to global epigenetic drift (de Cecco et al., 2013a, b, 2019; van Meter et al., 2014; Kaczkowski et al., 2016). Active TEs can affect gene expression by disrupting the promoter, enhancer, or gene body with new insertions or by introducing novel regulatory sequences (Inouye et al., 1984; Saigo, 1984; Saigo et al., 1984). Even without transposition, aberrant TE activation leads to transcriptome deregulation through the activation of neighboring genes via their strong promoters or the attraction of repressive chromatin modifiers and the spreading of repressive signatures across neighboring genomic regions. Therefore, enhancement of the heterochromatic silencing of repeat elements could be key to maintaining the healthspan. The gain-of-function effect of ectopic DNMT3L on TE repression observed in our current study was consistent with our previous DNMT3L loss-of-function study using *Dnmt3l-*KO embryo-derived MEFs (Liao et al., 2015). MEFs derived from *Dnmt3l-*KO embryos have an accelerated premature senescence phenotype, which is associated with a global reduction in repressive H3K9me3 and H3K27me3 markers (Liao et al., 2015). We further developed bioinformatic pipelines to quantify the representation of each TE subfamily from our published strand-specific RNA-seq datasets of *Dnmt3l−/−* and *Dnmt3l+/+* littermate-derived MEFs (Liao et al., 2015). We found that more than 90% of the differentially expressed TE subfamilies were upregulated in *Dnmt3l-*KO MEFs (Supplementary Table S1). These findings described above suggest that a long-term effect of DNMT3L-expressing progenitor cells can still impact a cell type with no detectable DNMT3L.

Not surprisingly, the expression of most of the tested genes increased after DNMT3L treatment, and the genes with enriched expression in young cells were found to be more involved in pathways related to proliferation. The pathway enriched with DNMT3L-affected genes that are derepressed during aging is less cataloged, and the stochastic errors that sporadically occur during cellular aging might be one explanation for this finding. Intriguingly, the transcription factors predicted from the set of genes that were downregulated after DNMT3L treatment and derepressed in old cells are involved in the pluripotency network. In stem cells, the pluripotent-associated proteins regulate autophagy (Sotthibundhu et al., 2018), and whether a similar regulation tends to self-rescue cells at the initiation of presenescence remains unclear. In addition to the pluripotency factor-target genes, TRP53-target genes were mostly upregulated in presenescent cells, expressed at lower levels in the young population and downregulated after DNMT3L treatment. TRP53 is best known as a tumor suppressor (Sotthibundhu et al., 2018) because it can either activate or repress genes. Intriguingly, we found that the expression of *Trp53* was sustained in DNMT3L-treated MEFs (Supplementary Figure S7), which indicated that the restored proliferation ability of DNMT3L-treated MEFs was unlikely due to the “silencing of Trp53” that is frequently found in cancers.

Although DNMT3L-treated MEFs exhibit a restored proliferative ability, the morphology of the DNMT3L-treated MEFs and the spindle-shaped young MEFs were not identical (Figure 1A). To provide clues regarding the cell identity of DNMT3L-treated MEFs, we performed a principal component analysis (PCA) to compare approximately 1000 gene expression profiles of other mouse cell lineages, including the immortalized MEF-base cell line 3T3, primary cultured somatic cells, stem cells, and other cancer cells described in a database (data not shown). The results showed that the transcriptomes of DNMT3L-treated MEFs were most similar to those of young MEFs. To further examine whether the DNMT3L-treated MEFs have fibrosis potential, we examined the expression levels of several representative fibrosis markers (Shih et al., 2018) from our microarray data (Supplementary Figure S10) and found no difference among young, old MEF and DNMT3L-treated MEFs in terms of their fibrogenic potential. The results indicated that the DNNMT3L pulse might have some effects on the transcriptional activity of certain extracellular matrix genes (*Col3a1* and *Ctgf*) without affecting markers for fibroblast activity (*Txndc5*, *Acta2*, and *Postn*). Our data showed that most of the fibrogenic protein genes were distinctly regulated in DNMT3L-treated MEFs. This suggested a limited role of DNMT3L in the process of tissue fibrosis. In addition, our transcriptomic data did not link the ectopic expression of *Dnmt3l* to cancer-related outcomes. The tumor formation assay in nude mice gave negative results, disproving a tumorigenic function for *Dnmt3l* in DNMT3L-treated MEFs (data not shown). The relationship between DNA methyltransferases (DNMTs) and tumorigenesis has been widely discussed (Zhang and Xu, 2017). Unlike DNMT1, DNMT3A and DNMT3B, no direct evidence has shown that DNMT3L contributes to carcinogenesis thus far. The current data are unlikely to link DNMT3L-treated MEFs to carcinogenesis, but we are not excluding the possibility that DNMT3L might lead to further unexpected cell fate changes beyond a repair of the chromatin state. Hopefully, our work could pave the way for the identification of more precise targets based on the DNMT3L-affected aging-associated network or downstream pathways associated with the resistance to senescence and prolongation of the cellular healthspan.

DNMT3L expression is rarely observed outside of germ cells and embryonic stem cell lineages. In MEFs from passage 1 to passage 8, DNMT3L expression, if any, was under the detection sensitivity for RNA-seq, RT-qPCR and Western, immunocytochemistry (Kao et al., 2014; Figure 5; unpublished observations). However, transient ectopic DNM3L expression induced long term effect observed in this study still provides potential physiological and pathological significance. For example, mouse DNMT3L has been demonstrated to be expressed in hematopoietic stem cells (Liu et al., 2013), and potentially have influence on the differentiated blood cell lineages (unpublished observation). In contrast, the Down syndrome associated overexpression of DNMT3L in neural progenitor cells of frontal cortex could underlie one mechanistic cause of the consequential neural disorder (Lu et al., 2016). It is also possible that aberrant transient DNMT3L expression may lead to detrimental effect for cells that need to be cleared out.

In summary, we discovered that transient DNMT3L expression halted senescence in aging/presenescent MEFs via enforcing global and regional chromatin surveillance. DNMT3L treatment of old MEFs restored their nuclear structure to a state closer to that of young cells by upregulating the nuclear envelope protein LaminB1 and histone H3. In addition, DNMT3L recruited the repressive epigenomic modifying complex DNMT3L-DNMT3A-KAP1-SETDB1-HDAC1 and increased H3K9me3 modifications in some ERVs and retrotransposons. DNMT3L also interacted with the PRC2 complex, resulting in increased H3K9me3 modification on a significant proportion of aging-associated derepressed single-copy genes (Figure 7). While current anti-aging research and applications place greater emphasis on eliminating senescent cells, we propose the possibility of minimizing the aging-associated relaxation of chromatin surveillance to prolong cellular health span.

## Experimental Procedures

### Animal Care and Cell Culture

The care of mice and the experimental procedures involving animals were approved by the Institutional Animal Care and Use Committee (IACUC) of National Taiwan University (approval number NTU-104-EL-00031 and NTU-105-EL-00123). Murine embryonic fibroblasts (MEFs) were derived from 13.5- to 14-day prenatal wild−type C57BL/6 mouse embryos and cultured with 5% CO_2_ and ambient oxygen (20%) in Dulbecco’s modified Eagle’s medium (DMEM) (Gibco, CA, United States) supplemented with 10% FBS, 100 U/ml penicillin and 100 μg/ml streptomycin.

### Transient Ectopic DNMT3L Expression

Mouse DNMT3L or control eGFP was cloned into a constitutive pCAG vector with neomycin resistance. Plasmid DNA was purified and transfected into MEFs using Lipofectamine 2000 (Thermo Fisher, CA, United States) according to the manufacturer’s instructions.

### Immunocytochemistry (ICC)

The cells were seeded onto poly-L-lysine slides (Thermo Scientific) and cultured overnight. After fixing in 4% PFA for 10 min at room temperature, the cells were washed twice with DPBS, treated with 0.5% Triton X-100 in PBS and blocked in 10% goat serum with 1–2% bovine serum albumin (BSA). After incubation with the primary anti-Ki67 antibody (ab16667), the sections and tubules were incubated with secondary antibodies (1:500 dilution, 715-485-150 DyLight 488 and 211-505-109 DyLight 549, Jackson ImmunoResearch), counterstained with Hoechst 33342 (Sigma) and mounted with mounting medium (Cat. P36934, Invitrogen).

### Western Blotting (WB)

Cells were homogenized and lysed with RIPA Buffer (Abcam) containing 1 mM phenylmethylsulfonyl fluoride (Sigma) and Protease Inhibitor Cocktail (Sigma) and centrifuged for 10 min at 16,000 × *g* at 4°C. The protein concentrations of the lysates from total cortical gray matter homogenates were determined by the bicinchoninic acid assay method (Pierce, Rockford, IL, United States). Lysates from equal amounts of cells were separated by sodium dodecyl sulfate–polyacrylamide gel electrophoresis (SDS−PAGE) and transferred to PVDF membranes (Millipore). The membranes were blocked with 5% BSA (Sigma) or 5% milk dissolved in PBST (PBS containing 0.1% Tween-20, Millipore) at room temperature for 2 h. The incubations with the primary antibodies at an appropriate dilution were performed at 4°C overnight with gentle shaking. The membranes were incubated with a horseradish peroxidase-conjugated secondary antibody (1:5000 dilution). The proteins were detected using a chemiluminescent reagent (Millipore) and a BioSpectrum Imaging System (UVP). The protein quantities were quantified by analyzing the images using ImageJ software^1^. The antibodies used for WB were anti−lamin B1 (ab16048), anti−α-tubulin (ab7291), anti−H3 (ab1791), anti-H3K9me3 (ab8898), anti-DNMT3L (E1Y7Q, Cell Signaling #13451), anti-GAPDH (ab181602), anti-SUZ12 (D39F6, Cell Signaling #37373) and anti-EZH2 (AC22, Merck Millipore #17-662) antibodies.

### Chromatin Immunoprecipitation (ChIP)

ChIP assays were performed using a LowCell# ChIP kit (Cat. Diagenode). Briefly, 1 × 10^6^ freshly collected cells were cross-linked using 1% formaldehyde for 8 min at room temperature, and the cross-linking was arrested with glycine. After centrifugation, the cells were resuspended in lysis buffer supplemented with a protease inhibitor cocktail (Cat. Diagenode) and 20 mM NaBu. The cross-linked chromatin was sheared to lengths of approximately 500 bp by ultrasound (Cat. Diagenode). The sheared chromatin samples were divided into 10 fractions, and each fraction incubated with 4 μg of anti-H3K4me3 (ab8895, Abcam), anti-H3K27me3 (07-449, Millipore) anti-FLAG (Cat. Sigma) or control IgGs (Cat. Diagenode) overnight at 4°C with rotation. The immunoprecipitated DNA was isolated using DNA isolation buffer (Diagenode), and specific genes in the purified DNA were amplified by qPCR and analyzed.

### Viral−Mediated Gene Transfer

FLAG-tagged *Dnmt3l* was flanked into the all-in-one inducible lentiviral backbone pAS4.1w.Ppuro-aOn vector using the *Nhe*I and *Eco*RV restriction enzyme cutting sites downstream of the TetOn operator. This constructed, inducible *Dnmt3l*-bearing plasmid and a pAS4.1w.Ppuro-aOn-RFP (control) plasmid were transfected, respectively, into 293T cells with TransIT-2020 transfection reagent (Mirus Bio) for the packaging of pseudotype lentivirus. The DOX-inducible lentiviruses produced were used for the infection of young MEFs. Young P2-P3 MEFs were seeded in 24-well plates at 70% confluence 1 day prior to infection. The MEFs were infected by viral suspension with fresh media containing 8 μg/ml polybrene. Puromycin selection was initiated at 5 μg/ml after 24 h of viral infection. After the non-viral infected MEFs were clearly killed, the selected infected MEFs were continuously cultured in selection medium with 2 μg/ml puromycin. When the number of Ki67-positive cells decreased to 20–30%, ectopic gene expression in the infected old MEFs was induced by treatment with 0.5 μg/ml doxycycline for 48 h, and the cells were then subjected to the following experiments. Additional details are supplied in the Supporting Information.

### Immunoprecipitation (IP)

The cells were lysed in NP40 buffer (Invitrogen) supplemented with 1x protease inhibitor cocktail (R1321, Fermentas) and 1 mM phenylmethylsulfonyl fluoride (PMSF). Immunoprecipitation was performed using a Dynabeads kit (Invitrogen) following the manufacturer’s recommended protocol. For IP, 50 μg of total protein from DNMT3L/RFP-expressing cells was used for each experiment. The antibodies used for IP were anti−FLAG (M2, Sigma #F3165) and anti−SUZ12 (D39F6, Cell Signaling #3737) antibodies.

### Total RNA Extraction and RT-qPCR

The cells were trypsinized from the culture dish and neutralized using an equal volume of culture medium containing 10% FBS. After two washes with DPBS, 500 g of the cells were pelleted in a 1.5-mL microcentrifuge tube and then centrifuged for 5 min at RT. Five hundred microliters of TRIzol^®^ reagent (Cat. 15596018, Invitrogen, Carlsbad, CA, United States) was added to 2 × 10^6^ cells, and the cells were then lysed by pipetting several times in the presence of TRIzol^®^ reagent and homogenized by 1 min of vortexing. RNA purification was accomplished using the RNeasy mini kit (Cat. 74104, QIAGEN, United States), and the RNA was then treated with RNase-Free DNase I Set (Cat. 79254, QIAGEN, United States) while bound to the RNeasy membrane and subjected to “On-Column DNase Digestion” for 30 min at room temperature. The DNA-free RNA was eluted in RNase-free water. For assessment of the total RNA quantity and quality, 1 μl of sample was loaded on a NanoDrop spectrophotometer (NP-1000, NanoDrop, Wilmington, DE, United States) and then subjected to 1% agarose gel electrophoresis or analysis with a bioanalyzer. The RNA was stored at −80°C, and the reverse transcription and quantitative polymerase chain reactions (RT-qPCRs) were performed using a SuperScript First-Strand Synthesis System (Invitrogen) and Roche LightCycler 480II instrument, respectively. The annealing temperature used for all the primers was 60°C (primers are listed in Supplementary Table S2).

### Statistics

The data are presented as the means ± standard errors of the mean (*SEMs*). The data analyses were performed using unpaired *t-*test or one−way analysis of variance (ANOVA) followed by *post hoc* Tukey’s test as appropriate. *p* < 0.05 was considered statistically significant.

## Data Availability Statement

All sequencing data are deposited at the GEO repository under the accession number GSE135258.

## Ethics Statement

The animal study was reviewed and approved by the National Taiwan University IACUC.

## Author Contributions

S-PL, YY, and H-FL conceived and planned the experiments. T-HK discovered the DNMT3L-mediated senescence-moderating phenomenon. YY, T-HK, H-FL, C-YY, J-YC, Y-TT, and H-TH performed the experiments. TH and C-CH performed the bioinformatic analysis. TH discovered the DNMT3L-mediated re-repression of PRC2-target genes in aging cells after DNMT3L treatment. MP and AM provided expertise and resources for transcriptome analysis of *Dnmt3l* KO MEFs. K-CY, Y-RC, and M-HT provided expertise, experimental support and critical comments on fibrosis, proteomic analysis and microarray analysis, respectively. All authors were involved in periodic discussion and critical feedback that helped to shape the research direction. S-PL provided overall funding and full-time supervision. YY and S-PL wrote the manuscript and incorporated comments from co-authors. YY created all the artwork (including graphic summary) in this manuscript.

## Conflict of Interest

The authors declare that the research was conducted in the absence of any commercial or financial relationships that could be construed as a potential conflict of interest.

## Footnotes

1 https://imagej.nih.gov/ij

## Abbreviations

ANOVA: analysis of variance
BSA: bovine serum albumin
ChEA: ChIP Enrichment Analysis
ChIP: chromatin immunoprecipitation
co-IP: coimmunoprecipitation
DEGs: differentially expressed genes
DMEM: Dulbecco’s modified Eagle’s medium
DNMTs: DNA methyltransferases
DNMT3L: DNA methyltransferase 3-like
DOX: doxycycline
ERVs: endogenous retroviruses
HDAC: histone deacetylases
IP: Immunoprecipitation
KO: knockout
MEFs: mouse embryonic fibroblasts
PCA: principal component analysis
PMSF: phenylmethylsulfonyl fluoride
PRC2: polycomb repressive complex 2
RFP: red fluorescent protein
RT-qPCRs: reverse transcription and quantitative polymerase chain reactions
SASP: senescence-associated secretory phenotype
SDS-PAGE: sodium dodecyl sulfate–polyacrylamide gel electrophoresis
SEMs: standard errors of the mean
TE: transposable element
WB: Western blot.
