# Supplementary material for: Transient DNMT3L Expression Reinforces Chromatin Surveillance to Halt Senescence Progression in Mouse Embryonic Fibroblast
Source: Front Cell Dev Biol. 2020 Mar 4;8:103. doi: 10.3389/fcell.2020.00103 (PMC7064442; doi:10.3389/fcell.2020.00103)
Supplement: Supplementary file 1 [file Data_Sheet_1.PDF]

Supporting Information

FIGURE S1

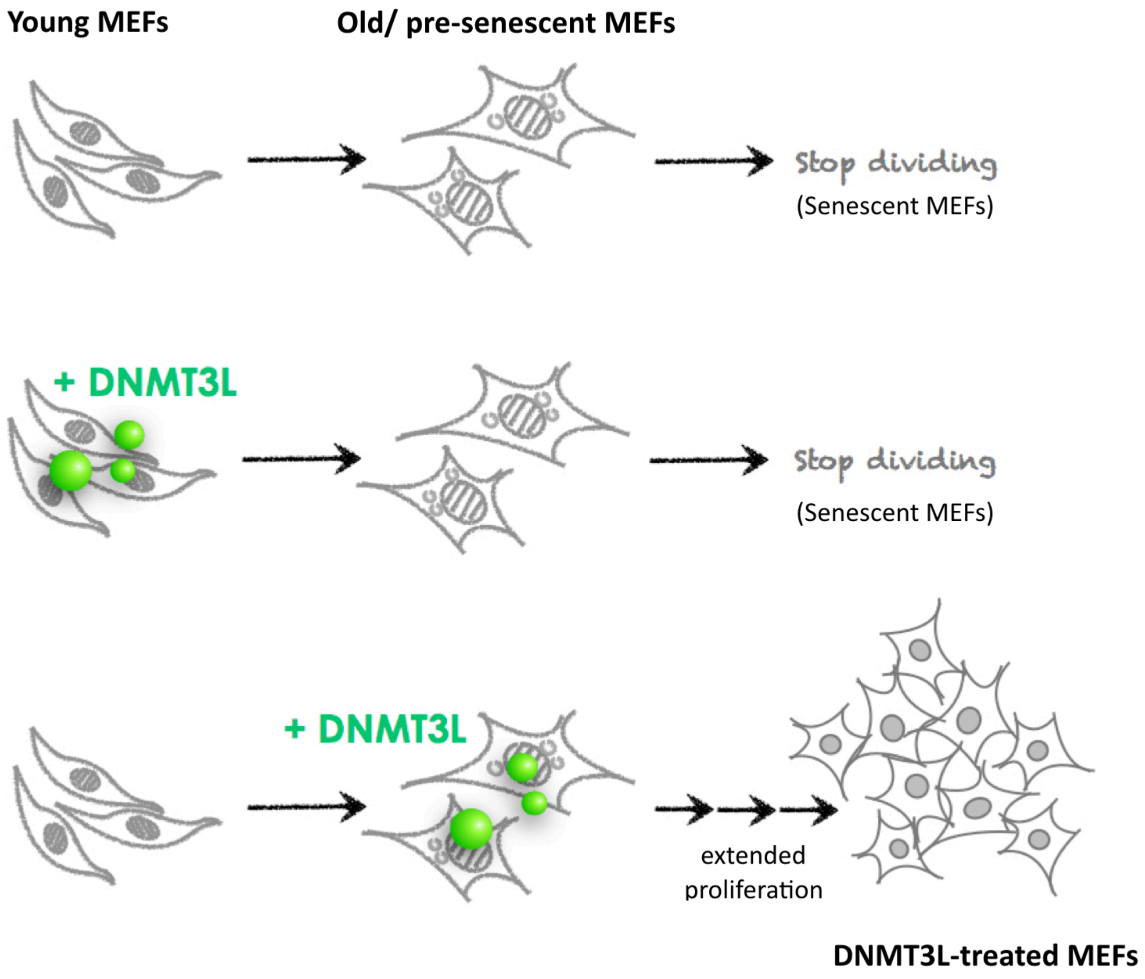

Fig. S1. Illustration of the effective timing of ectopic Dnmt3l expression for extending cell proliferation. The expression of Dnmt3l only in “sufficiently old MEFs” could halt premature senescence. The ectopic expression of Dnmt3l in MEFs was performed in MEFs at different passages. We determined the effective expression timing of DNMT3L based on whether the transient DNMT3L treatment of cells at a specific passage could prolong the cell-doubling period compared with parallel controls. To minimize the bias of defining the “passage numbers” from independent biological replicates during cell culture, we standardized the ectopic expression window of Dnmt3l among different batches of experiments using the Ki67 index (Ki67-positive staining indicates actively dividing cells). With prolonged passaging, the percentage of Ki67-positive cells gradually decreased. We found that transfection at passages 5 to 8 (corresponding to 20-30% Ki67-positive MEFs in the population) represented the optimal timing of Dnmt3l expression for extending the proliferation activity of MEFs (to observe a halt in senescence progression).

Supporting Information

FIGURE S2a

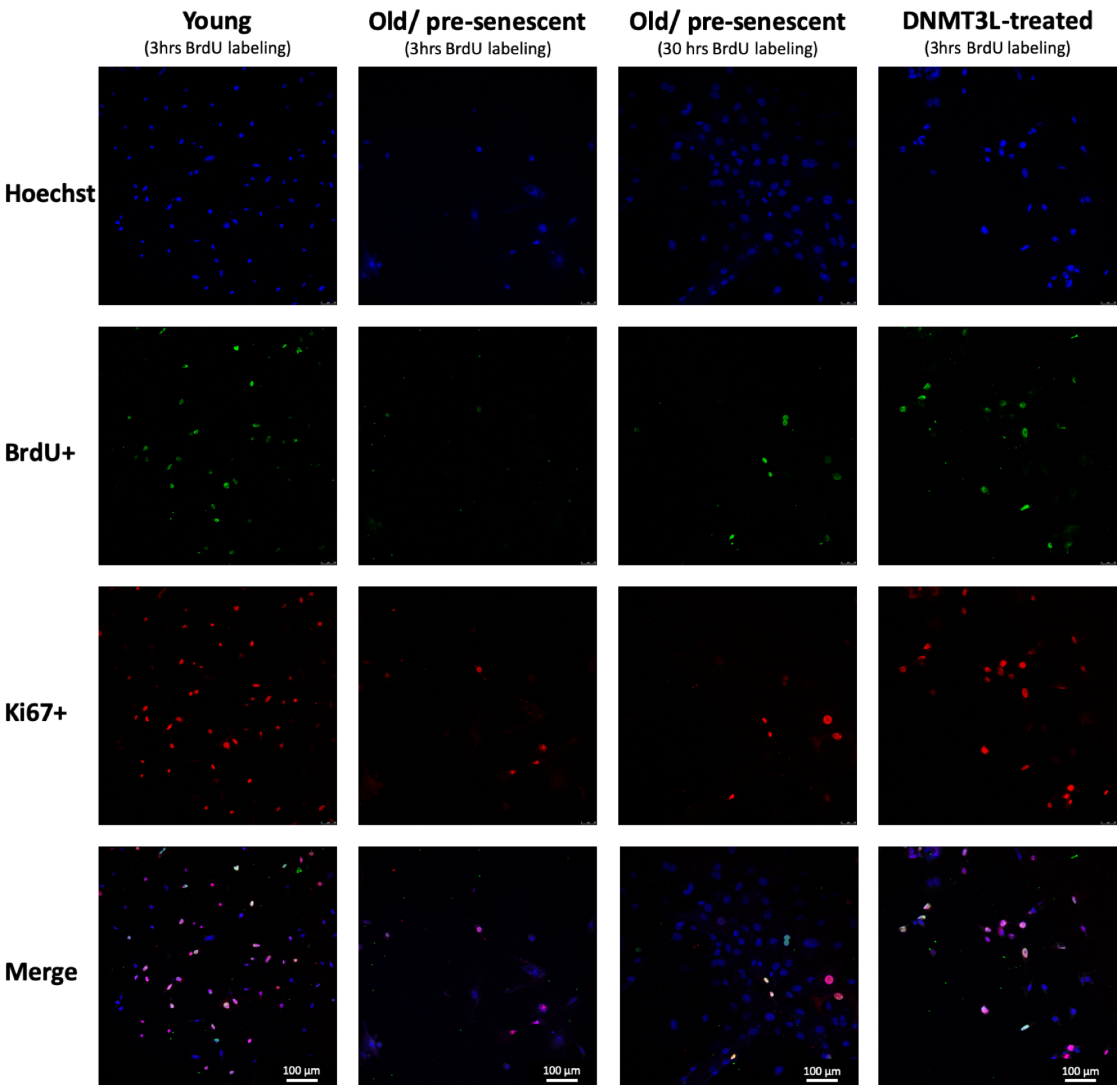

Supporting Information

FIGURE S2b

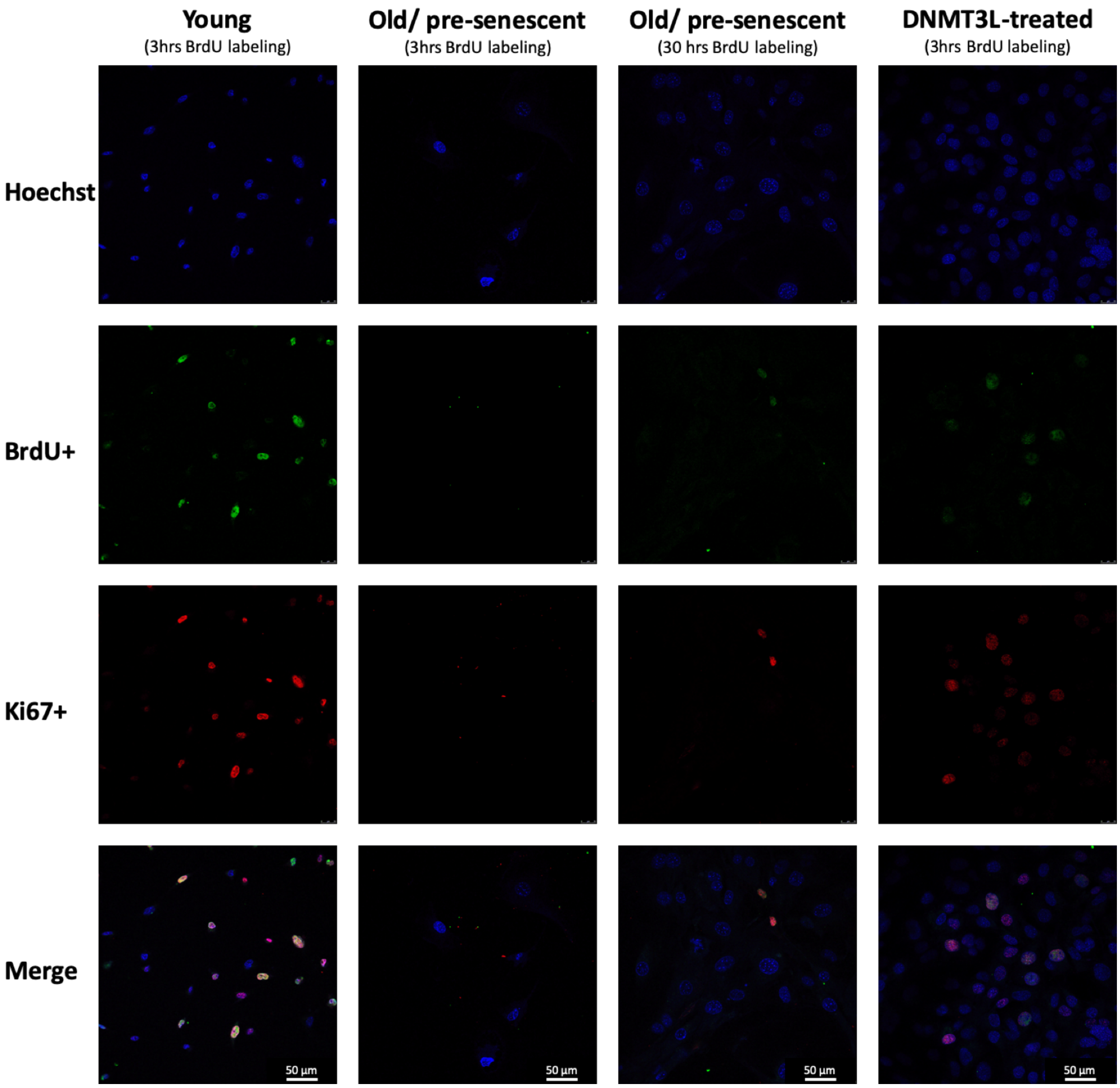

FIGURE

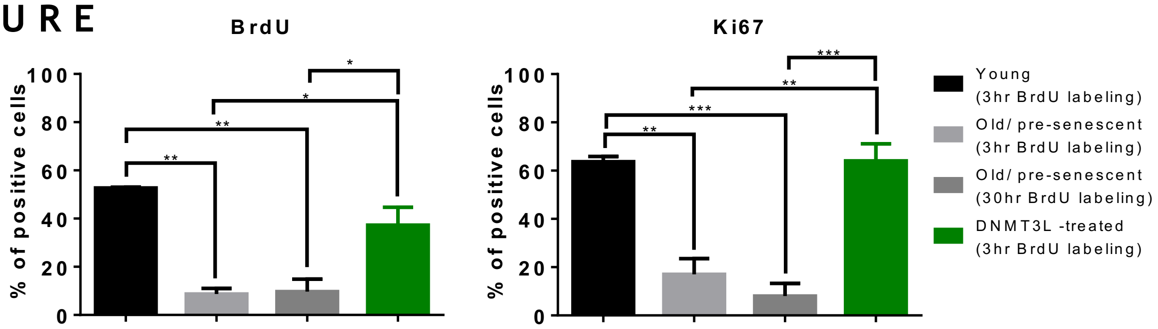

(One-way ANOVA with Tukey's comparison test,
p≤0.05*; ≤0.01**; ≤0.001*** and ≤0.0001****)

Supporting Information

Fig. S2. a-b Cells seeded on coverslips were pulse labeled with 25 μM BrdU under normal culture condition for 3 hr or long-term treated for 30 hr as indicated. The treated cells were fixed in 4% PFA for 20 min, followed by hydrolysis with 2N HCl for 30 min at room temperature to denature cellular DNA, wherein incorporated BrdU can be exposed for detection. After permeabilization by 0.5% Triton-X 100 for 5 min at room temperature, the cells were simultaneously incubated with the primary antibodies anti-BrdU (ab136650; mouse) and anti-Ki-67 (ab16667; rabbit) (both from Abcam, Cambridge, UK); and then the secondary antibodies against mouse and rabbit IgG, conjugated with CF 488A and CF 594, respectively (both from Biotium, Hayward, USA), and counterstained with Hoechst 33342 (Sigma, St. Louis, USA) for DNA. **a-b** Double staining of Ki-67 and BrdU in young, old/pre-senescent and DNMT3L-treated cells. (scale bar indicated)

Fig. S2. c Analysis with the double staining micrographs of BrdU treated cells were done for the frequency of BrdU or Ki-67 positive cells from at least three separate fields. One-way ANOVA with Tuckey's comparison test was used to determine the specific difference in the phenotype of the groups. ($p<0.05$*; $p<0.01$**; $p<0.001$***)

Supporting Information

FIGURE S3

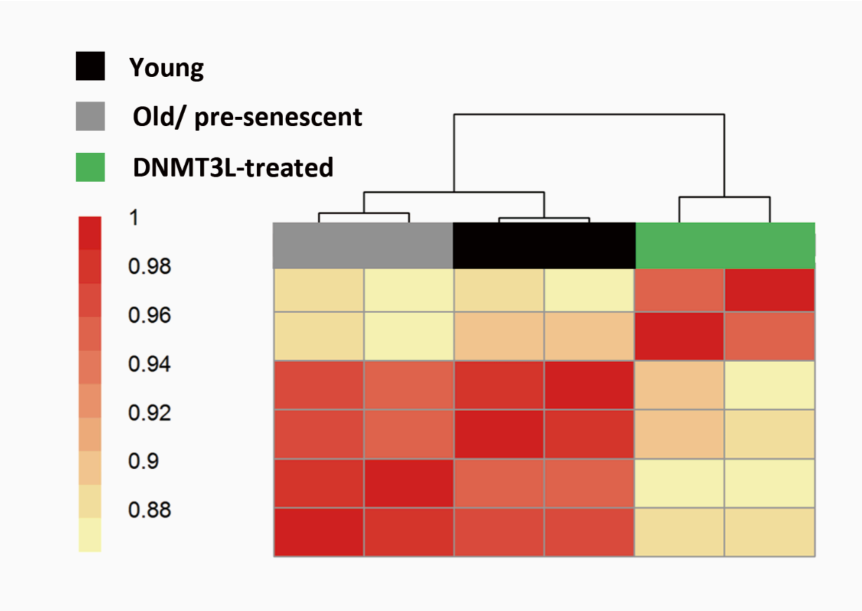

Fig. S3. Pairwise Spearman correlation matrix of annotated gene expression patterns of young, old/presenescent and DNMT3L-treated MEFs determined by a microarray assay.

Supporting Information

FIGURE S4

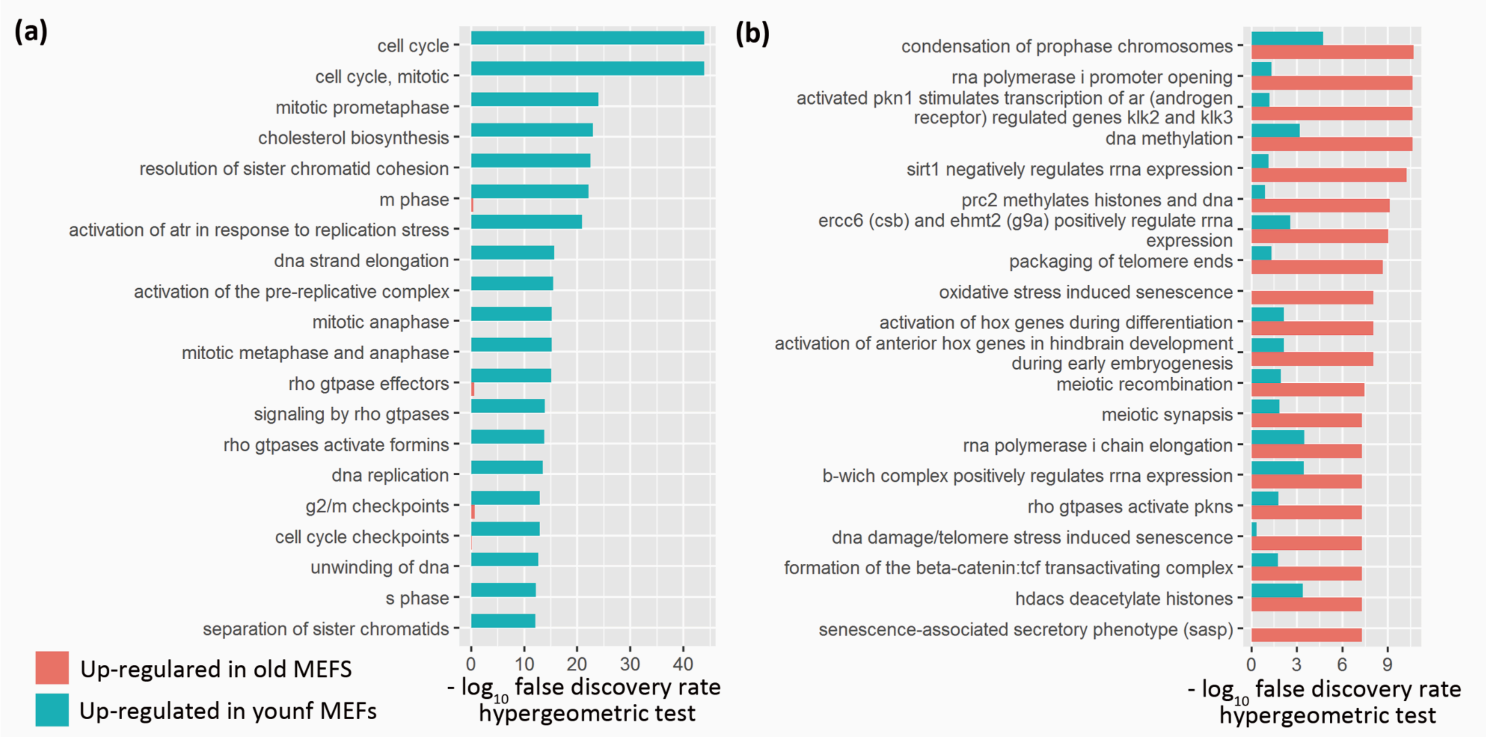

Fig. S4. (a) Gene ontology (GO) analysis of genes upregulated in young MEFs. (b) GO analysis of genes upregulated in old MEFs. The significance of the enriched GO term was indicated by the -log10 false discovery rate of the hypergeometric test.

Supporting Information

FIGURE S5

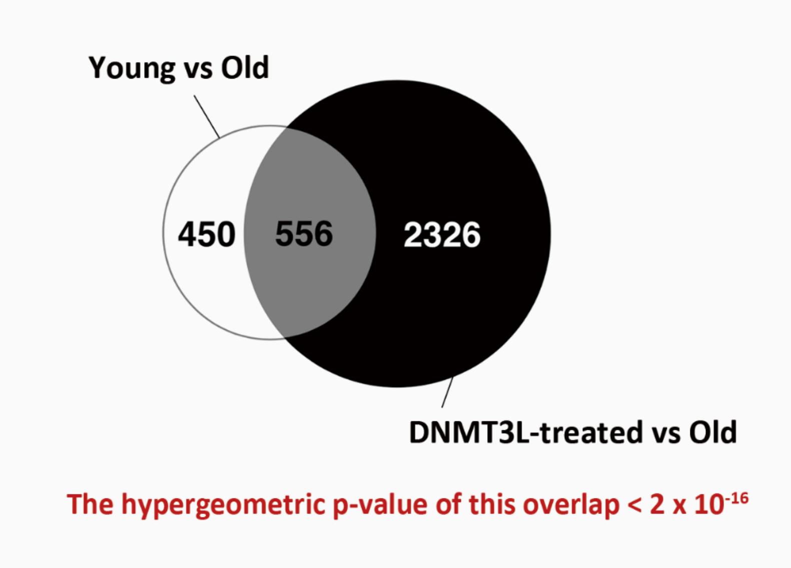

Fig. S5. Differentially expressed genes (DEGs) between young and old MEFs and their intersection with DEGs before and after the DNMT3L pulse. The hypergeometric p-value of this overlap was less than $2x10^{-16}$.

Supporting Information

FIGRE S6

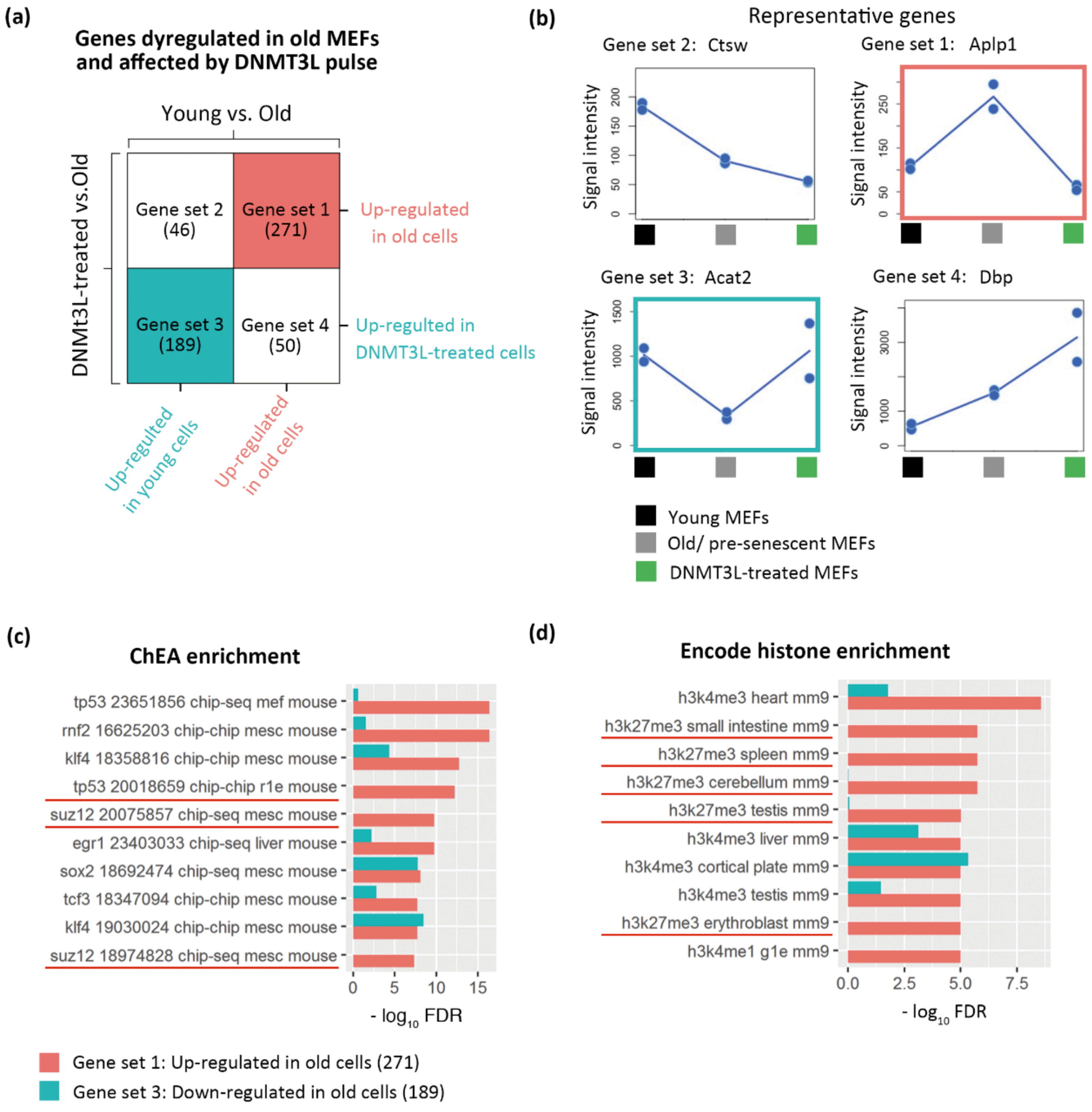

Fig. S6. (a) We classified 556 of the genes that were differentially expressed between young and old MEFs and altered after the DNMT3L pulse (the intersection shown in Fig. S3) into four gene sets based on genes that were up- or downregulated in each comparison ("young vs old MEFs" and "DNMT3L-treated vs old MEFs"). The genes were classified as follows based on their expression in young, old, and DNMT3L-treated MEFs: the genes in gene set 1 (271) were upregulated in old MEFs compared with young MEFs and downregulated in DNMT3L-treated MEFs compared with old MEFs; the genes in gene set 2 (46) were downregulated in old MEFs compared with young MEFs and in DNMT3L-treated MEFs compared with old MEFs; the genes in gene set 3 were downregulated in old MEFs compared with young MEFs and upregulated in DNMT3L-treated cells compared with old MEFs; and the genes in gene set 4 were upregulated in old MEFs compared with

young MEFs and in DNMT3L-treated MEFs compared with old MEFs. As expected, the 556 deregulated genes were affected by DNMT3L treatment (differentially expressed genes between DNMT3L-treated and old cells). (b) Representative genes from the gene sets described in Fig. S4a. (c) The intersection of genes that were downregulated in old MEFs compared with either young MEFs or DNMT3L-treated MEFs and were initially downregulated in the old/presenescent population (gene set 3) were enriched in the cell-cycle and mitotic-related pathways, whereas the genes that were affected by DNMT3L treatment and were upregulated in the old/presenescent population (gene set 1) were enriched in signal transduction, extracellular matrix organization, and cell-to-cell communication. Intriguingly, a relatively higher proportion of genes enriched in gene set 1 are TP53-, pluripotent factor-, or PRC2 member-targeted genes. (d) The output of Encode Histone Enrichment for gene set 1 was characterized by repressive histone modification (H3K27me3).

Supporting Information

FIGURE S7

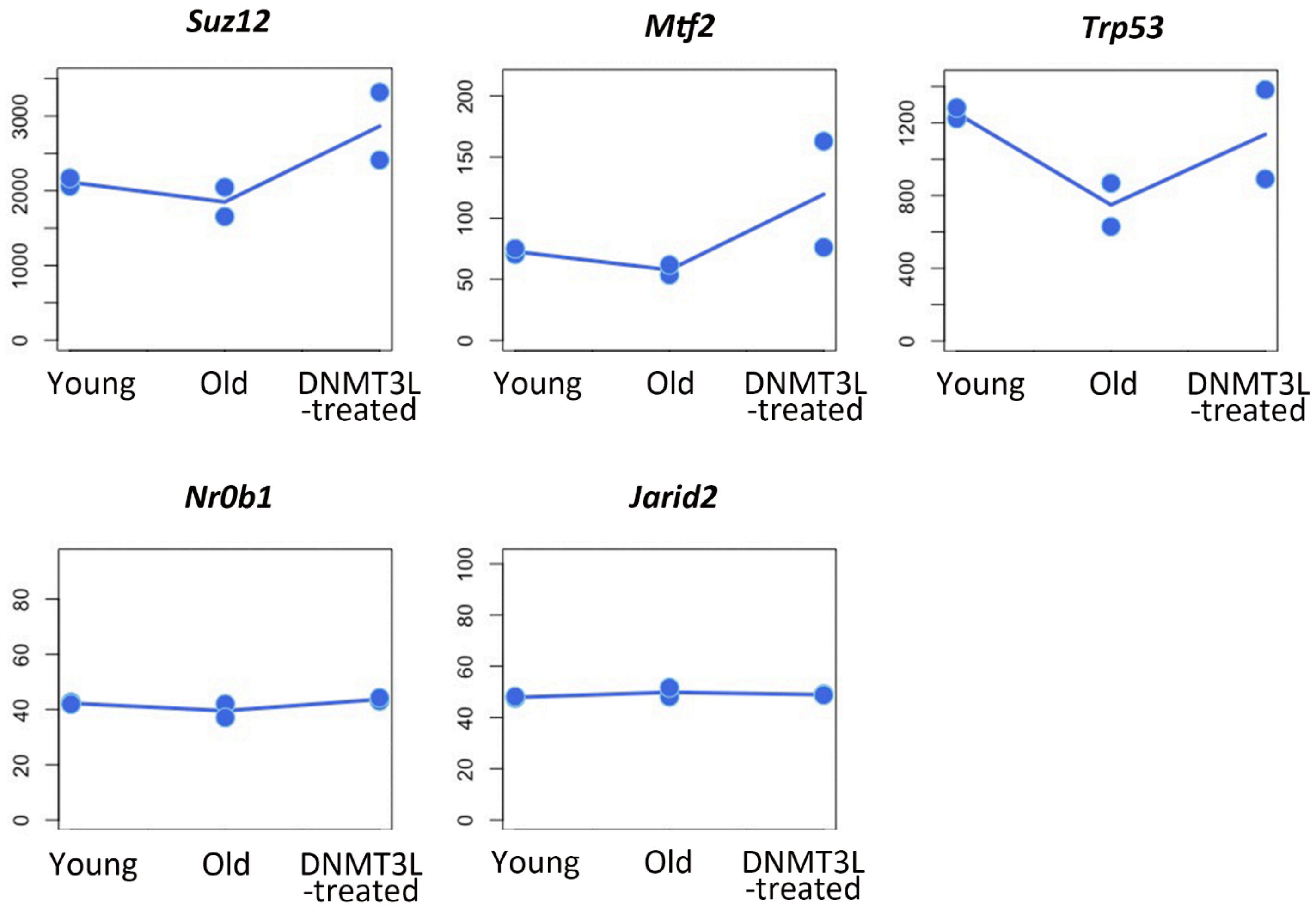

Fig. S7. Microarray data showing the transcription levels of Suz12, Mtf2, Trp53, Nr0b1 and Jarid2 in young, old/presenescent and DNMT3L-treated MEFs.

Supporting Information

FIGURE S8

(a)

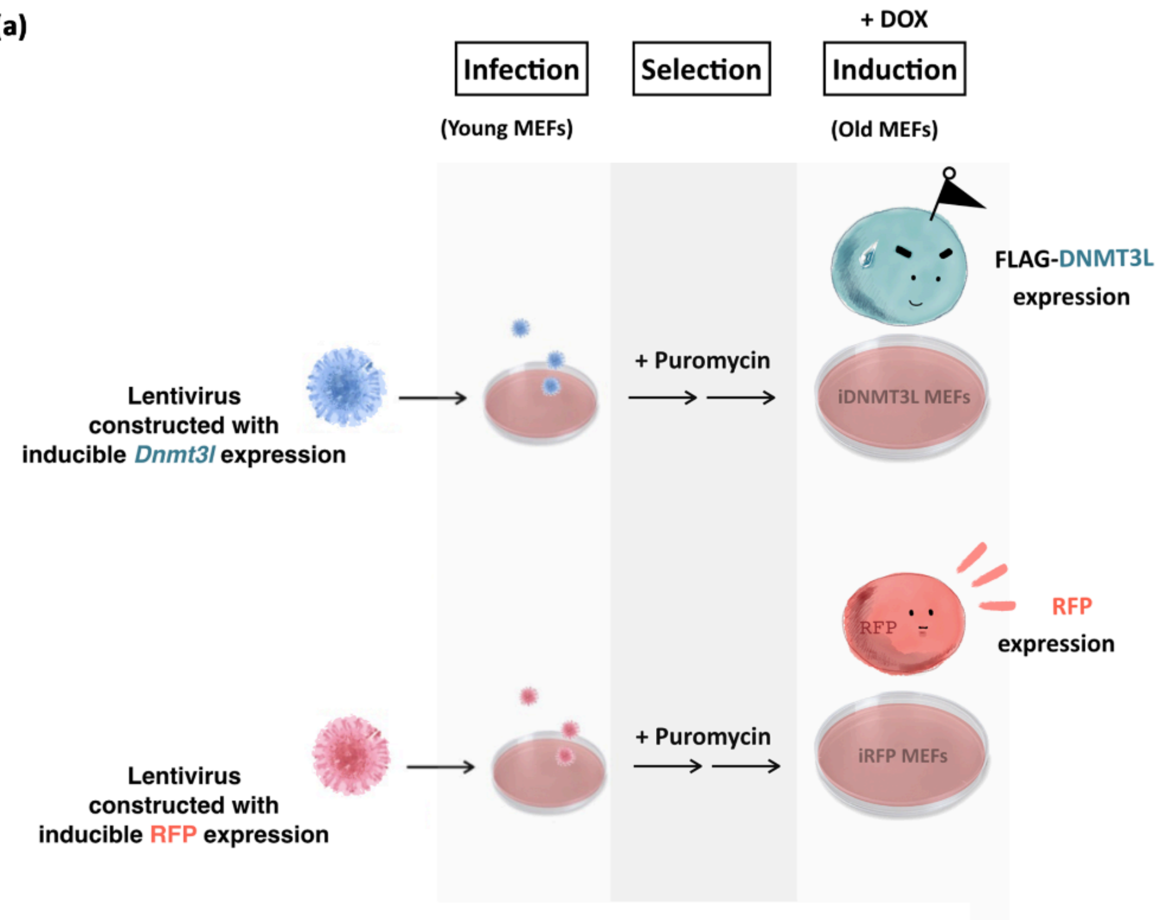

(b)

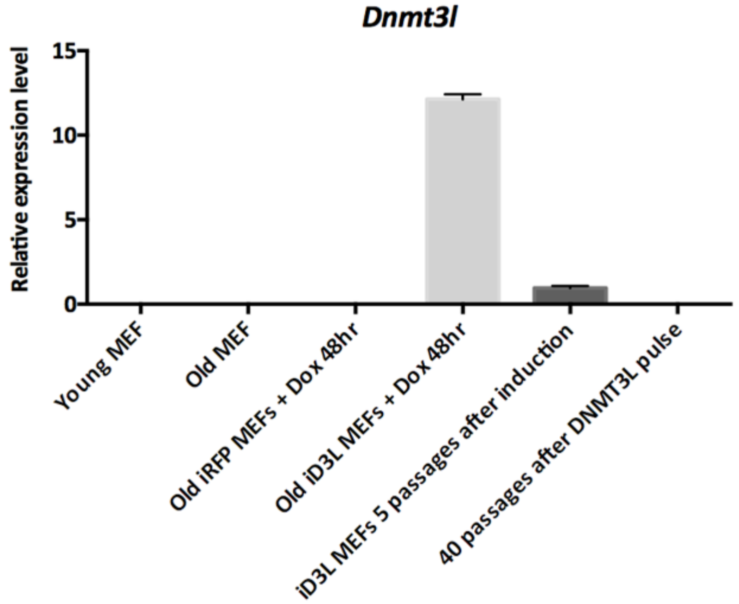

Fig. S8. (a) Illustration of the viral-infected inducible tag-Dnmt3l expression system in MEFs. Young MEFs were infected with doxycycline-inducible lentivirus containing the tag-Dnmt3l sequence (iD3L-lentivirus) or RFP-coding sequence (iRFP-lentivirus) in parallel. We termed the MEFs infected with iD3L-lentivirus "iD3L MEFs", whereas the MEFs infected with the iRFP-lentivirus were denoted "iRFP MEFs". Puromycin selection was

continuously maintained until doxycycline induction. (b) RNA expression levels of Dnmt3l in each sample obtained by RT-qPCR detection.

Supporting Information

FIGURE S9

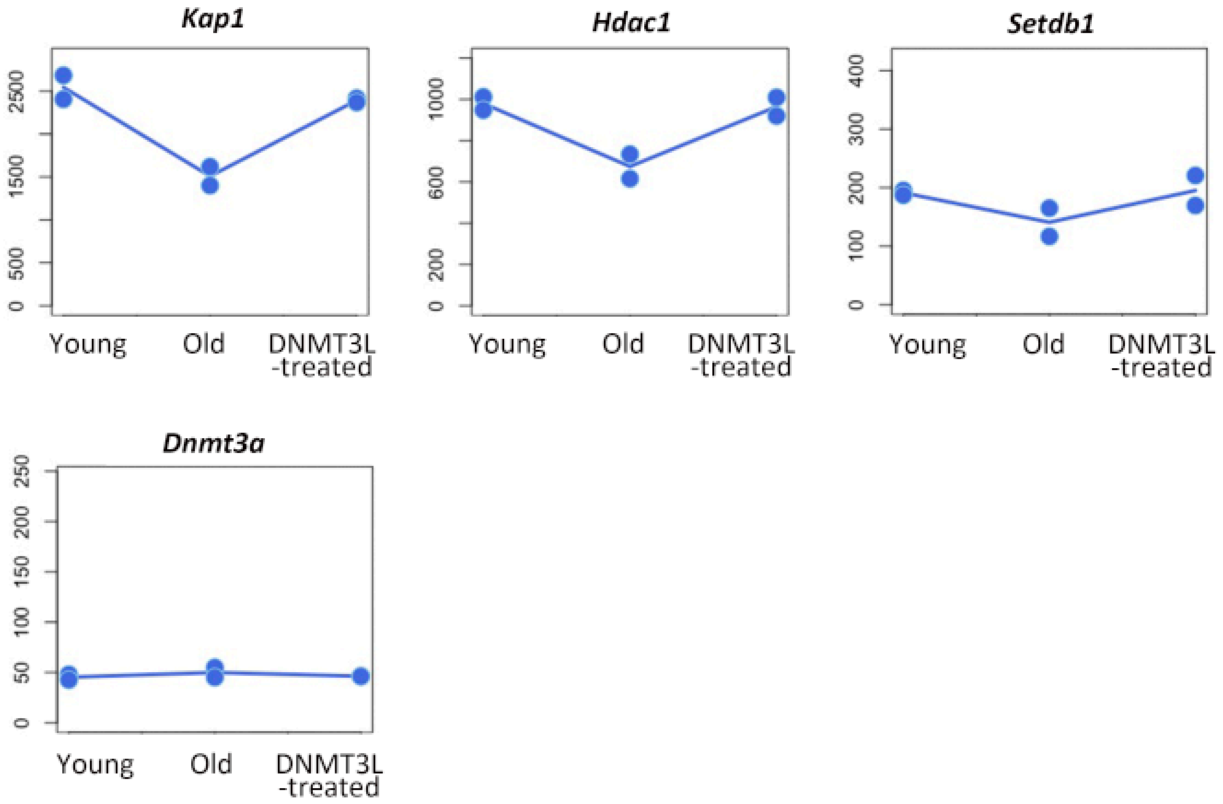

Fig. S9. Microarray data showing the transcription levels of *Kap1*, *Hdac1*, *Setdb1* and *Dnmt3a* in young, old/presenescent and DNMT3L-treated MEFs.

Supporting Information

FIGURE S10

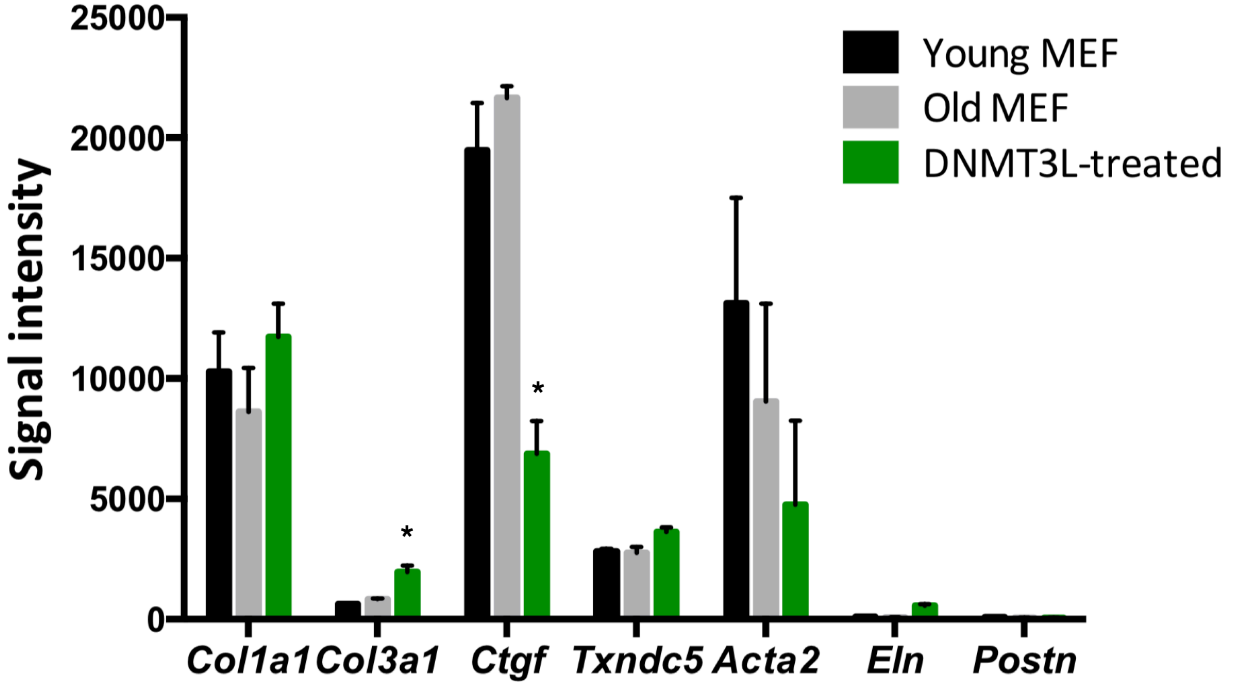

Fig. S10. The relative expression of fibrosis-related genes (± SEM) in young, old and DNMT3L-treated MEFs according to microarray. Representative fibrosis-related genes: *Col1a1, Col3a1, Ctgf, Txndc5, Acta2, Eln and Postn*.
