# Supplementary material for: Transient DNMT3L Expression Reinforces Chromatin Surveillance to Halt Senescence Progression in Mouse Embryonic Fibroblast
Source: Front Cell Dev Biol. 2020 Mar 4;8:103. doi: 10.3389/fcell.2020.00103 (PMC7064442; doi:10.3389/fcell.2020.00103)
Supplement: Supplementary file 2 [file Table_1.pdf]

Table S1. TE subfamilies showing differential expression between *Dnmt3l* -/- and *Dnmt3l* +/+ littermate-derived MEFs. The expression quantity is indicated as transcript per million reads (tpm) according to previously published strand-specific RNA sequencing results. The sequences have been deposited in the NCBI GEO dataset under accession number GSE69007.

Differentially expressed TEs	Dnmt3l +/+ MEF replicate 1 (tpm)	Dnmt3l +/+ MEF replicate 2 (tpm)	Dnmt3l -/- MEF replicate 1 (tpm)	Dnmt3l -/- MEF replicate 2 (tpm)	*p* value
LTR/ERVK:RLTR31D_MM	333.784	370.379	508.313	466.997	0.02008459
LTR/ERV1:RLTR4_Mm	147.444	162.873	214.387	235.817	0.020674749
DNA/TcMar:X4b_DNA	107.633	164.962	280.231	231.739	0.044483942
LTR/ERVL-MaLR:ORR1A3	129.068	146.372	194.55	206.059	0.018210771
LTR/ERV1:RLTR6C_Mm	122.123	134.683	161.163	169.223	0.026445141
LTR/ERVK:MurERV4-int	97.537	107.926	130.687	125.79	0.042119698
LTR/ERVK:RMER17C2	89.012	80.467	125.116	112.879	0.027248853
LINE/L1:L1MdTf_II	48.887	53.296	66.15	72.479	0.0260158
LINE/L1:L1MdGf_II	29.179	31.898	42.694	47.529	0.02777258
DNA/hAT-Blackjack:MER81	30.145	27.307	36.475	40.492	0.034413192
DNA/hAT-Charlie:MER58D	21.705	23.567	32.146	35.512	0.024260586
LTR/ERV1:RLTR30D2_MM	17.217	21.724	33.107	27.864	0.044245715
LINE/L2:X15_LINE	13.57	18.357	30.307	26.501	0.030105904
LINE/CR1:X17_LINE	20.65	22.371	27.497	26.481	0.02458434
LTR/ERVK:RMER17A	17.659	19.92	24.624	26.066	0.026990023
LTR/ERV1:RLTR14_RN	18.138	19.825	27.174	25.076	0.018549331
LINE/L1:L1MdF_II	17.669	19.821	22.209	24.365	0.048228577
LINE/L1:L1M	14.3	16.713	21.711	22.803	0.036255343
DNA/hAT-Charlie:Charlie10	16.326	15.905	22.577	21.59	0.013544301
LTR/ERV1:LTR37A	12.142	14.578	18.947	21.297	0.028714823
LINE/RTE-X:L4_B_Mam	14.844	14.249	19.238	20.361	0.015158771
LTR/ERVK:RLTR16B_MM	11.073	11.118	17.155	18.192	0.024841149

LTR/Gypsy:MamGypLTR1b	9.767	11.765	19.927	17.588	0.018461026
LTR/ERVK:RLTR20A	11.07	9.497	16.163	17.301	0.013828051
LTR/ERVK:ETnERV2-int	11.059	12.627	16.749	17.094	0.042596592
LTR/ERVL-MaLR:MTE2b-int	8.172	9.972	14.598	14.833	0.047997039
LTR/ERVK:RLTR9A3	11.409	11.222	12.177	12.616	0.044535423
LTR/ERVK:RLTR16C_MM	8.235	8.308	13.028	12.433	0.019815904
DNA/TcMar-Tigger:Tigger20a	7.702	7.315	10.616	10.438	0.008687081
LTR/ERV1:LTRIS_Mm	6.731	7.923	10.444	9.321	0.044776647
DNA/hAT-Tip100:MER45C	5.943	5.099	8.328	9.074	0.015494942
DNA/hAT-Tip100:Zaphod	6.384	6.398	8.321	8.973	0.045617176
DNA/hAT-Charlie:Charlie11	4.983	5.741	7.356	8.471	0.038408336
DNA/hAT-Tip100:MamTip3	4.678	5.334	6.612	7.467	0.03513994
DNA/hAT-Blackjack:MER94B	5.386	4.745	7.635	7.452	0.031926614
DNA/hAT-Charlie:Charlie31a	4.439	4.43	6.63	7.262	0.039814232
LTR/ERVK:RLTR10B2	5.731	5.367	6.241	6.705	0.047449442
LINE/L1:L1MEj	3.991	4.345	6.296	6.678	0.006299328
LTR/ERVL:MER76	3.912	3.464	5.329	5.866	0.017105527
DNA:Eutr11	2.59	3.141	6.116	5.698	0.007834686
LTR/ERVL-MaLR:MLT1B-int	4.177	3.615	5.623	5.425	0.040362808
LTR/Gypsy:MamGypLTR4	2.982	4.103	6.152	5.424	0.048120764
LTR/Gypsy:LTR81B	4.241	4.258	4.541	4.65	0.046452142
DNA/TcMar:DNA1_Mam	2.046	2.869	4.165	4.593	0.042573281
LTR/ERVL:MER70B	3.269	3.411	3.795	4.044	0.04056836
LTR/ERVK:RLTR19B	2.538	2.921	4.584	4.005	0.029627935
LTR/ERV1:RLTR30D_RN	1.271	1.261	3.689	3.127	0.041478518
LTR/ERVK:ERVB5_1-I_MM	1.014	0.563	2.776	2.348	0.014781246
LTR/ERVK:ERVB4_3-I_MM	1.052	0.957	1.614	1.812	0.02470933
LTR/ERVL:LTR69	1.176	1.089	1.419	1.318	0.036808296

LTR/ERVK:MMERVK10D3_LTR	0.604	0.897	1.482	1.214	0.047928931
LTR/Gypsy:LTR81AB	0.189	0.107	0.526	0.651	0.019217846
LTR/ERVK:RLTR13D	0.272	0.197	0.484	0.41	0.028167049
LTR/ERVL-MaLR:MLT1H-int	21.11	19.514	12.639	13.157	0.023990743
LTR/ERVK:IAPA_MM-int	1.659	2.003	1.116	0.799	0.032735721
LTR/ERV1:RLTR1E_MM	3.713	4.176	1.57	0.754	0.022843573
LTR/ERVL-MaLR:MLT1I-int	1.807	1.438	0.764	0.551	0.033808569
