# Supplementary material for: Transient DNMT3L Expression Reinforces Chromatin Surveillance to Halt Senescence Progression in Mouse Embryonic Fibroblast
Source: Front Cell Dev Biol. 2020 Mar 4;8:103. doi: 10.3389/fcell.2020.00103 (PMC7064442; doi:10.3389/fcell.2020.00103)
Supplement: Supplementary file 3 [file Table_2.pdf]

Table S2. Primer list.

Primer ID	5'-3' sequence
Class I ERV: ERV U3 (F)	CCC CAA ATG ACC GAG AAA TA
Class I ERV: ERV U3 (R)	GCG GTT ACA GAA GCG AGA AG
Class I ERV: eMLV (F)	GTA AAA ACT CCA CAC TCG GC
Class I ERV: eMLV (R)	ACG ATT CGG ATG CAA ACA GC
ClassII ERV: IAP (F)	CGT GAG AAC GCG TCG AAT AA
ClassII ERV: IAP (R)	TTC TGG TTC TGG AAT GAG GG
ClassII ERV: MMERVK-10C (F)	CAA ATA GCC CTA CCA TAT GTC AG
ClassII ERV: MMERVK-10C (R)	GTA TAC TTT CTT CTT CAG GTC CAC
ClassIII ERV: Mu-ERVL (F)	ATC TCC TGG CAC CTG GTA TG
ClassIII ERV: Mu-ERVL (R)	AGA AGA AGG CAT TTG CCA GA
ClassIII ERV: MAT (F)	ATG TCT TGG GGA GGA CTG TG
ClassIII ERV: MAT (R)	AGC CCC AGC TAA CCA GAA CT
Dnmt3l (F)	GGAACTCTCCAGGTGTAC
Dnmt3l (R)	GTGTCCATCATCATCATACAG
Rplp0 (F)	ATC TGC TGC ATC TGC TTG
Rplp0 (R)	CGA CCT GGA AGT CCA ACT AC
Aplp1(F)	GTGGGCGTCTAACCCTTCAC
Aplp1(R)	CTGCGGGTCCAGAAGACAG
Dnajc6(F)	TGAAAATAAAGGTGCCTCGTCTC
Dnajc6(R)	TCAGGTTACTGAATAGCCTCCC
Kcnj4(F)	ATGCACGGACACAACCGAAA
Kcnj4(R)	CTGGGACTTGTTGCTCAGG
Sim2 (F)	CCTTCTGACCACGACGAGATG
Sim2 (R)	TCGAAGAAAGAAAGACCGCTCTA
Aplp1 promoter (F)	CTTCCCCATCCCCAGGAC
Aplp1 promoter (R)	ACTGTCATTGCTGCTTCTGC
Dnajc6 promoter (F)	GACCTGGCATTCTGGTGACT
Dnajc6 promoter (R)	CAGCTCTATCCCCAACCAAA
Kcnj4 promoter (F)	CTTGCTTGCGCTCTCTCTCT
Kcnj4 promoter (R)	TTGATGACGGCGTTTGTAAC
Sim2 promoter-exon1(F)	CGGGTCTGCCATAAACAAAC
Sim2 promoter-exon1 (R)	GCTTCCTTAGTCCGGTCGTC
